# Polyvalent Glycan Functionalized Quantum Nanorods as Mechanistic Probes for Shape-Selective Multivalent Lectin-Glycan Recognition

**DOI:** 10.1021/acsanm.2c05247

**Published:** 2023-03-14

**Authors:** James Hooper, Darshita Budhadev, Dario Luis Fernandez Ainaga, Nicole Hondow, Dejian Zhou, Yuan Guo

**Affiliations:** † School of Food Science and Nutrition and Astbury Centre for Structural Molecular Biology, University of Leeds, Leeds LS2 9JT, United Kingdom; ‡ School of Chemistry and Astbury Centre for Structural Molecular Biology, University of Leeds, Leeds LS2 9JT, United Kingdom; § School of Chemical and Process Engineering, University of Leeds, Leeds LS2 9JT, United Kingdom

**Keywords:** multivalent interaction, quantum rod, glycoconjugate, geometry, FRET, electron microscopy

## Abstract

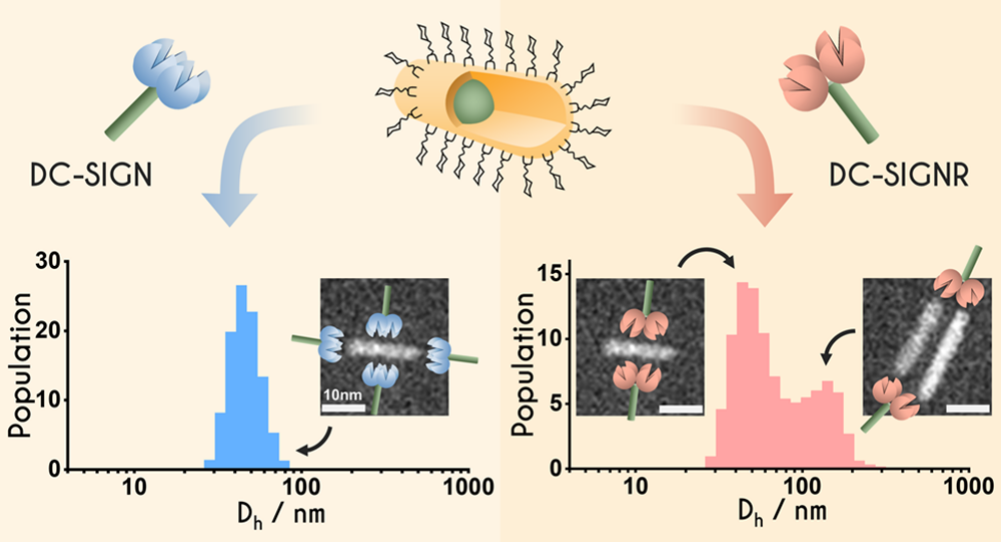

Multivalent lectin-glycan interactions (MLGIs) are widespread in biology and hold the key to many therapeutic applications. However, the underlying structural and biophysical mechanisms for many MLGIs remain poorly understood, limiting our ability to design glycoconjugates to potently target specific MLGIs for therapeutic intervention. Glycosylated nanoparticles have emerged as a powerful biophysical probe for MLGIs, although how nanoparticle shape affects the MLGI molecular mechanisms remains largely unexplored. Herein, we have prepared fluorescent quantum nanorods (QRs), densely coated with α-1,2-manno-biose ligands (QR-DiMan), as multifunctional probes to investigate how scaffold geometry affects the MLGIs of a pair of closely related, tetrameric viral receptors, DC-SIGN and DC-SIGNR. We have previously shown that a DiMan-capped spherical quantum dot (QD-DiMan) gives weak cross-linking interactions with DC-SIGNR but strong simultaneous binding with DC-SIGN. Against the elongated QR-DiMan, DC-SIGN retains similarly strong simultaneous binding of all four binding sites with a single QR-DiMan (apparent *K*_d_ ≈ 0.5 nM, ∼1.8 million-fold stronger than the corresponding monovalent binding), while DC-SIGNR gives both weak cross-linking and strong individual binding interactions, resulting in a larger binding affinity enhancement than that with QD-DiMan. S/TEM analysis of QR-DiMan-lectin assemblies reveals that DC-SIGNR’s different binding modes arise from the different nanosurface curvatures of the QR scaffold. The glycan display at the spherical ends presents too high a steric barrier for DC-SIGNR to bind with all four binding sites; thus, it cross-links between two QR-DiMan to maximize binding multivalency, whereas the more planar character of the cylindrical center allows the glycans to bridge all binding sites in DC-SIGNR. This work thus establishes glycosylated QRs as a powerful biophysical probe for MLGIs not only to provide quantitative binding affinities and binding modes but also to demonstrate the specificity of multivalent lectins in discriminating different glycan displays in solution, dictated by the scaffold curvature.

## Introduction

Research into the effects of nanomaterial surface chemistry and scaffold design in biology has led not only to key advances in the ever-growing field of nanomedicine but also to the discovery of novel tools to answer important biological questions.^1−4^ The exploration of fine-tuning nanoparticle chemistry to achieve biological functionality has been extensively investigated for over four decades; however, the study of a nanoparticle’s geometric design elements, such as size and shape, and their mechanistic influences in biology remains in its infancy. Though some studies have shown that varying nanoparticle size and shape can have a strong effect on various biological processes such as cell uptake,^5−7^ endocytic pathways,^8−10^ cytokine/antibody production,^8,11−14^ or pathogen inhibition,^15−17^ the relationship between nanoparticle geometry and biological function remains a fairly under-explored topic for a lot of processes. Moreover, often little has been done to elucidate the specific mechanistic differences caused by shape variation at the molecular level, such as binding affinities or binding modes, which are essential for identifying how nanomedicines work and, perhaps more importantly, for guiding the design of more effective therapeutics.

Multivalent lectin-glycan interactions (MLGIs) are widespread and play a pivotal role in pathogen infection, immune regulation, and cell–cell communication.^18^ It is thus unsurprising that glycan-displaying nanomaterials have been widely exploited for potential antiviral and immuno-therapeutic applications due to simple polyvalent glycan functionalization. For glyconanomaterials, the geometric parameters, such as size and shape of nano-scaffolds, inevitably affect their surface glycan display. As natural lectins are often multimeric, variations in glycan display may lead to changes in the number of binding sites that can be occupied at one time, which dictates the strength and mode of lectin-glycan binding and, subsequently, biological function. A large number of glyco-nano-scaffolds with different geometries have been employed to target lectins. These include spherical scaffolds (e.g., inorganic nanoparticles, fullerenes, dendrimers, hyperbranched polymers, polymersomes, etc.),^19−29^ tubular/rod-shaped structures (e.g., gold nanorods, carbon nanotubes, and cylindrical micelles),^7,17,30−33^ planar sheets (e.g., graphene nanosheets),^32,34^ and structures that are less well-defined (e.g., linear polymers)^16,35−39^ or more complex in shape.^7,33,40−42^ Since these results are obtained with scaffolds of different glycan composition, softness, size, shape, and glycan density, it is difficult to directly compare results from one another to draw general conclusions. While studies have shown that the scaffold size and shape can affect their lectin binding and pathogen inhibition properties,^7,8,15−17,31,33,43^ the molecular mechanisms underlying such differences remain unclear. For example, Kikkeri’s group found that, among three differently shaped mannosylated gold nanoparticles (e.g., spheres, stars, and rods), gold nanorods consistently exhibited stronger bacterial binding than the other two shapes, although no characterization of the molecular mechanisms behind such differences was presented.^7,17,33^ Consequently, new tools are needed in order to identify the rationales behind these differences, allowing us to better understand the molecular mechanisms of shape dependency for such interactions. In this regard, probes that can reveal how scaffold shape affects MLGI binding mode and affinity are highly valuable, allowing us to establish a geometric design rule for glycan-nanoparticles for potent and specific targeting of MLGIs for therapeutic application.

We have recently developed densely glycosylated fluorescent quantum dots (glycan-QDs) as new mechanistic probes for MLGIs.^21,22^ These glycan-QDs were able to not only quantify MLGI affinity *via* a ratiometric QD-sensitized dye Förster resonance energy transfer (FRET) readout but also dissect the exact binding modes and affinity enhancing mechanisms of MLGIs *via* hydrodynamic size analysis and STEM imaging of lectin binding-induced glycan-QD assemblies.^21,22^ Here, two closely related, immunologically important tetrameric glycan-binding viral receptors, DC-SIGN and DC-SIGNR (collectively denoted as DC-SIGN/R hereafter),^44,45^ were used as model lectins due to their differences in viral transmitting properties despite sharing 77% amino acid identity, the same tetrameric architecture and identical monovalent mannose-binding motifs.^46^ Using QDs bearing α-1,2-manno-biose (DiMan)-glycans (QD-DiMan) as probes, we were able to extract key structural and mechanistic information for DC-SIGN/R related MLGIs. We revealed that, although both lectins bound multivalently with QD-DiMan, it was achieved through different binding modes, resulting in very different affinities. DC-SIGN was found to simultaneously bind to the same QD-DiMan with all four carbohydrate-recognition domains (CRDs), giving rise to strong binding (apparent equilibrium binding dissociation constant, *K*_d_, 2.1 ± 0.5 nM). However, DC-SIGNR was found to cross-link between QD-DiMan particles, with a much weaker affinity (*K*_d_ ≈ 633 nM, ∼300-fold weaker than that of DC-SIGN).^21^ Additionally, DC-SIGN binding, monitored *via* FRET, was detected at very low protein-to-QD molar ratios (PQRs) and increased linearly with the PQR till the QD surface was fully saturated with protein. However, for DC-SIGNR, due to the low affinity and cross-linking binding nature, saturation occurred at a much higher PQR and significant binding was only observed as the PQR was increased above a certain threshold.

Compared to spherical QDs, the elongated quantum nanorods (QRs) have a higher extinction coefficient and single particle brightness, which is highly beneficial for fluorescence-based applications.^47−49^ Moreover, its optical properties can be tuned by changing not only the particle size but also the aspect (length-to-width) ratio. This feature can be highly beneficial for some applications, e.g., bioluminescence resonance energy transfer (BRET), where an aspect ratio of 3 has been shown to give the highest BRET efficiency.^50,51^ Furthermore, the distinct curvatures between the central cylindrical section and spherical ends make the QR an excellent platform for studying how scaffold geometry controls MLGI properties by displaying glycans polyvalently on the QR. For such glycan-QRs, the glycan displays at the ends will closely resemble those of spherical glycan-QDs, whereas, in the middle, the glycans will be presented more like a curved plane wrapped around the center of the nanorod. Therefore, each glycan-QR presents two distinct glycan displays, allowing us to probe their different effects on MLGIs using the same glycan-nanoparticle.

**Figure 1:**
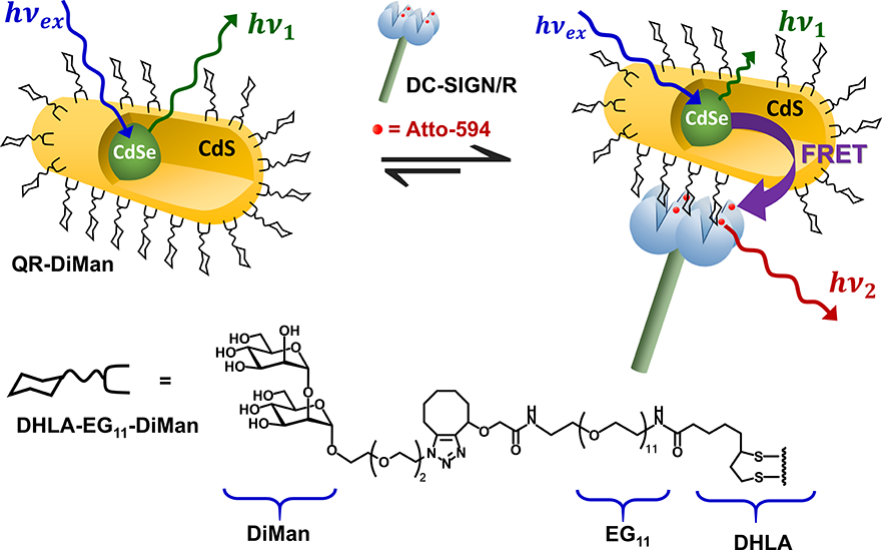
Schematic depicting the FRET readout for binding of an excited QR scaffold capped with DHLA-EG_11_-DiMan ligands (chemical structure depicted beneath) with DC-SIGN labeled with an Atto-594 dye molecule.

Here, we have densely coated a CdSe/CdS based QR with DHLA-EG_11_-DiMan ligands to make the first glycan-QR probe for analyzing the shape dependency of DC-SIGN/R-related MLGIs at the molecular level in solution (Figure 1). By developing a QR-FRET-based ratiometric MLGI affinity readout, together with hydrodynamic size and S/TEM analysis of lectin binding-induced QR assemblies, we show that the distinct glycan display curvatures of QR-DiMan can effectively discriminate MLGI properties between DC-SIGN and DC-SIGNR. DC-SIGN binds strongly and simultaneously to one QR-DiMan regardless of surface curvature with sub-nM *K*_d_, comparable to that of QD-DiMan. In contrast, DC-SIGNR binds simultaneously to the glycans displayed on the central cylindrical section of the same QR but cross-links between glycans displayed on the spherical ends of different QRs. Thus, the QR’s unique geometry and its strong fluorescence and high electron microscopy contrast have allowed us to reveal how glycan-nanoparticle surface curvature affects the solution-phase binding mechanisms of target MLGIs at the molecular level.

## Results and Discussion

### QR-DiMan Preparation and Characterization

A CdSe/CdS core/shell (dot in a rod) QR with maximal emission at λ_EM_ ∼ 560 nm (denoted as QR_560_ hereafter) was chosen to construct the QR-DiMan *via* our previously described ligand conjugation method.^53^ QR_560_ was chosen due to its similar core diameter to the QD scaffolds used in our previous study,^21^ allowing for direct comparison of results. A dihydrolipoic acid-undeca(ethylene glycol)-α-1,2-manno-biose (DHLA-EG_11_-DiMan; Figure 1; SI, Section 2.1)-based multifunctional ligand was synthesized as described previously.^21^ Each ligand contains three unique functional domains: a DHLA group for strong QR surface anchoring,^53^ a flexible EG_11_ linker for imposing high water solubility, stability, and resisting non-specific interactions,^54,55^ and a terminal DiMan group for specific lectin binding. QR-DiMan was produced by performing cap-exchange using a deprotonated DHLA-EG_11_-DiMan ligand in a homogeneous CHCl_3_/MeOH/EtOH solution, giving rise to high cap-exchange efficiency as described previously for QD-DiMan.^21,56^ QR-DiMan was found to be relatively compact, with a mean hydrodynamic diameter (*D*_h_) of 20.8 ± 4.8 nm, obtained by DLS, and a mean core length and diameter of 14.7 ± 5.7 and 3.1 ± 0.7 nm, respectively, obtained by S/TEM imaging (see SI, Figure S14; all size data reported here are mean ± 1/2 full width half maximum, FWHM). The first excitonic absorption and emission peaks were observed at 541 and 560 nm, respectively, and the fluorescence quantum yield (QY) was determined as 6.2%. This represents a significant reduction from its nominal QY of ∼68% prior to cap-exchange. This result agrees well with the literature: CdSe/CdS-based QDs and QRs have shown to display significantly reduced fluorescence QY after cap-exchange.^46,49^ By calculating the difference between the amount of ligand added and that which remained in the supernatant post cap-exchange, *via* phenol-sulfuric acid carbohydrate quantification, the number of DHLA-EG_11_-DiMan ligands capped on each QR was estimated as 370 ± 30 (see SI, Section 2.2).^20−22^

### Binding Affinity Determination *via* FRET

The QR’s strong fluorescence was exploited as a ratiometric FRET readout to quantify the binding affinity between QR-DiMan and acceptor fluorophore-labeled DC-SIGN/R. Lectin labeling was achieved by coupling a maleimide-Atto-594 dye *via* the site-specifically introduced Q274C and R287C mutations in DC-SIGN and DC-SIGNR, respectively. These labeling sites lie close to, but not in, the glycan binding pockets, enabling us to obtain sufficient FRET signals without inhibiting the lectins’ glycan binding properties, as confirmed previously.^21,22,57^ The QR-Atto-594 FRET pair has good spectral overlap and a respectable Förster radius *R*_0_ of 4.8 nm (see SI, Section 2.3), ensuring that efficient FRET between QR-DiMan and labeled lectins can happen upon binding. Meanwhile, there is little overlap of their emission spectra, allowing for straightforward separation of the donor and acceptor fluorescence without the need of spectral deconvolution.^57^

**Figure 2:**
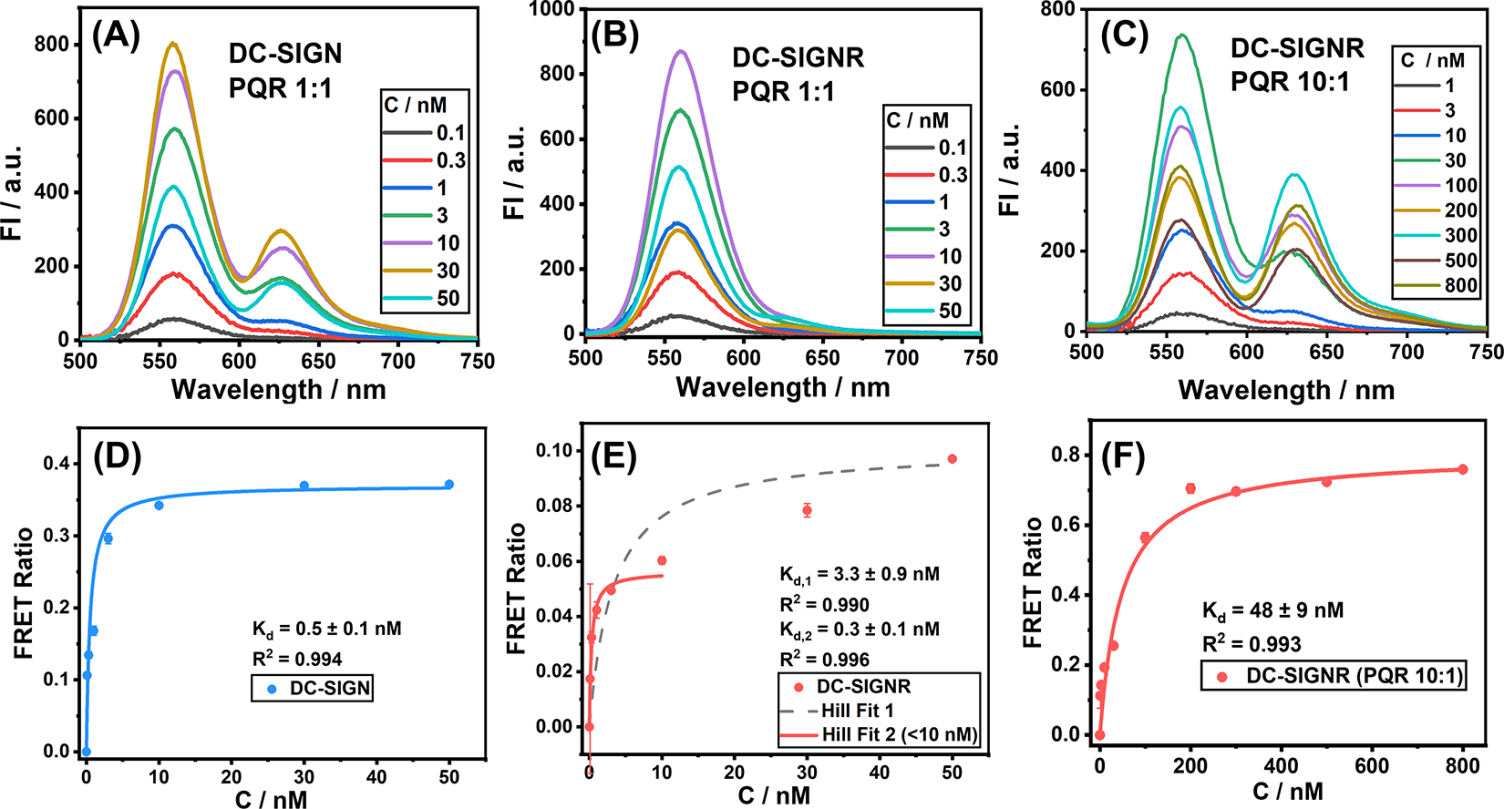
Direct excitation background-corrected fluorescence spectra of varying concentrations of QR-DiMan after binding with (A) DC-SIGN (PQR = 1), (B) DC-SIGNR (PQR = 1), or (C) DC-SIGNR (PQR = 10). The corresponding FRET ratio–concentration (*C*) relationships and Hill fits (eq 1) for QR-DiMan binding with (D) DC-SIGN (PQR = 1), (E) DC-SIGNR (PQR = 1), or (F) DC-SIGNR (PQR = 10). (Fitting parameters are summarized in Table 1; *R*^2^ ≥ 0.99).

The affinity assays were carried out by mixing QR-DiMan with labeled lectins under different concentrations but under a fixed protein–QR molar ratio (PQR) of 1:1 in a binding buffer (20 mM HEPES, 100 mM NaCl, 10 mM CaCl_2_, pH 7.8, containing 1 mg/mL bovine serum albumin to reduce non-specific adsorption). The resulting fluorescence spectra were recorded using a fixed excitation wavelength (λ_EX_) of 450 nm, corresponding to the absorption minimum of the Atto-594 acceptor to reduce the dye direct excitation background. Exciting an equilibrated mixture of QR-DiMan with labeled lectins resulted in fluorescence of unbound QR (peaking at ∼559 nm) or, if binding occurred, energy transfer *via* FRET from the excited QR donor to the Atto-594 acceptor, giving rise to simultaneously quenched QR and enhanced Atto-594 fluorescence (at ∼627 nm, Figure 1). These fluorescence spectra were corrected by subtracting dye direct excitation background spectra of the labeled lectins, without QR-DiMan, under identical conditions (Figure 2A–C). The resulting dye-to-QR fluorescence intensity ratio (apparent FRET ratio)–concentration relationship was then fitted by the Hill equation to extract the apparent binding *K*_d_ values (eq 1; where *F* is the FRET ratio, *I* is the peak emission intensity, [P] is the protein concentration, *n* is the Hill coefficient, and *K*_d_ is the apparent equilibrium binding dissociation constant).^21,57^ Here, *n* = 1 was assumed because most binding assays were measured under a PQR of 1:1, under which most QRs should be bound by just a single lectin, and hence there should be no positive or negative influence of QR-bound lectins toward further binding of lectins to the same QR-DiMan. As the FRET ratio is proportional to the fraction of protein bound to the QR under a fixed PQR of 1:1,^21^ this method is robust and can provide accurate affinity measurement of both strong and weak MLGIs.^57^
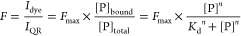
1

The FRET ratio–concentration relationship for DC-SIGN binding with QR-DiMan was fitted very nicely (*R*^2^ > 0.99) by the Hill equation and revealed a *K*_d_ of 0.5 ± 0.1 nM at a PQR of 1:1 (Figure 2D; Table 1). This represents a massive 1.8 million-fold enhancement of affinity (β) over the corresponding DiMan-CRD monovalent binding (*K*_d_ = 0.9 mM) and a per-glycan-normalized affinity enhancement (β/*N*) of ∼4900 (Table 1).^52^ Interestingly, this affinity is ∼4-fold as strong as that of QD-DiMan·DC-SIGN binding, studied previously (*K*_d_ = 2.1 ± 0.5 nM).^21^ The sub-nanomolar *K*_d_, here, demonstrates the high suitability of QR-DiMan for potent DC-SIGN targeting. The difference in affinity between QR-DiMan and QD-DiMan in DC-SIGN binding could be due to subtle changes in the inter-glycan distances and/or glycan display curvatures, allowing the former to have better spatial and/or orientation match with DC-SIGN’s four binding sites to form stronger binding than the latter.

**Table 1:** Summary of Fitting Parameters Obtained from the FRET Ratio–Concentration Relationship for QR-DiMan Binding with DC-SIGN and DC-SIGNR (*R*^2^ > 0.99 for All Fits)

*protein*	*PQR*	*F_max_*	*K_d_^QR^(nM)*	*β*^a^	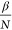	*K*_*d*_^*QD*^^b^	*K*_*d*_^*QD*^/*K*_*d*_^*QR*^^b^
DC-SIGN	1:1	0.37 ± 0.01	0.5 ± 0.1	∼1,800,000	∼4900	2.1 ± 0.5	4
DC-SIGNR	1:1^c^	0.056 ± 0.003	0.3 ± 0.1	∼3,000,000	∼8100	633 ± 77	2110
	1:10	0.80 ± 0.03	48 ± 9	∼19,000	∼51		13

a β is the enhancement factor over the monovalent interaction (i.e., *K*_d_^mono^/*K*_d_^QR^), *K*_d_^mono^ = 0.9 mM for CRD-DiMan binding.^52^

b *K*_d_^QD^ is the equilibrium binding dissociation constant between QD-DiMan and DC-SIGN (PQR 1:1) or DC-SIGNR (PQR 10:1), as reported previously.^21^*K*_d_^QD^/*K*_d_^QR^ signifies the enhancement in *K*_d_ for the binding of QR-DiMan over that of QD-DiMan.

c Only low concentration data points of ≤10 nM are included in the fit.

The FRET ratio for DC-SIGNR binding is considerably lower than that of DC-SIGN at PQR = 1 under equivalent conditions, implying a weaker binding compared to the former. This result is fully consistent with that of DC-SIGN/R binding with QD-DiMan reported previously.^16^ The overall FRET ratio–concentration relationship for DC-SIGNR binding with QR-DiMan could be fitted by the Hill equation (*R*^2^ = 0.990), giving an apparent *K*_d_ of 3.3 ± 0.9 nM (gray broken line), although several data points were found to deviate considerably from the fitting curve (Figure 2E). The resulting FRET ratio–concentration relationship appeared to display biphasic binding behavior, where the concentration dependency of the FRET ratio exhibits a secondary increase at higher concentrations. By fitting only the first few data points at the low concentration range (i.e., ≤ 10 nM), a good fit (*R*^2^ > 0.996) with an apparent *K*_d_ of 0.3 ± 0.1 nM was obtained (Figure 2E). This *K*_d_ value is comparable to that of DC-SIGN, suggesting that a similar interaction is taking place, likely involving the same degree of binding multivalency (e.g., binding of all four CRDs to the same QR-DiMan). The appearance of the broader (slower increasing signal) secondary binding phase suggests that further binding can occur with the increasing concentration *via* a relatively weak binding interaction. This may indicate the formation of cross-linking between QR-DiMan·DC-SIGNR assemblies, in a similar binding mode to that observed previously with QDs.

In order to obtain a more accurate overall binding affinity between DC-SIGNR and QR-DiMan, the binding assay was performed using a PQR of 10:1, which significantly improved the FRET signals (Figure 2C). Fitting the resulting FRET ratio–concentration relationship using eq 1 gave an apparent binding *K*_d_ of 48 ± 9 nM (Figure 2F), which is ∼13-fold stronger than that of the QD-DiMan·DC-SIGNR binding under equivalent conditions (*K*_d_ = ∼633 nM, PQR = 10).^21^ The binding affinity enhancement for DC-SIGNR is more substantial than that for DC-SIGN, which is likely due to the presence of the additional high affinity 1:1 binding component observed with DC-SIGNR under a PQR of 1.

### Binding Mode Determination *via* FRET and Hydrodynamic Size Analysis

**Figure 3:**
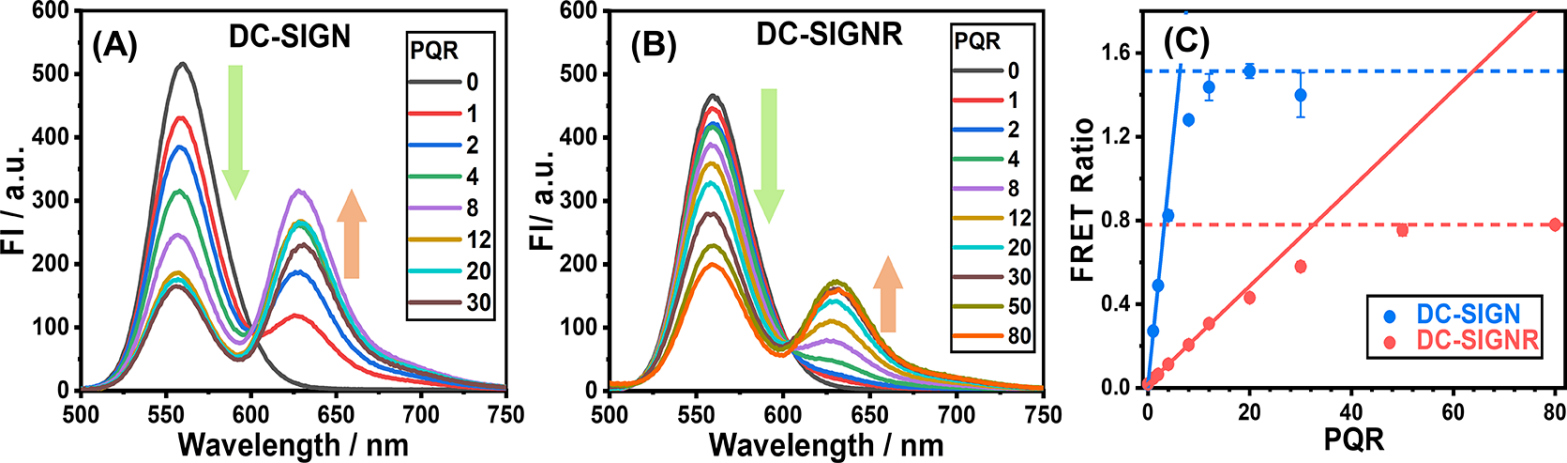
Direct excitation background-corrected fluorescence spectra corresponding to titration of (A) DC-SIGN or (B) DC-SIGNR against a fixed concentration of QR-DiMan (10 nM). (C) Plots of the corresponding FRET ratio–PQR relationships fitted with linear fits of the initial PQR data points (solid line; PQR ≤ 4 or 12 for DC-SIGN and DC-SIGNR, respectively). The fits give intercept = 0.018 ± 0.002 and 0.0194 ± 0.0006 and slope = 0.24 ± 0.01 and 0.0234 ± 0.0002 for DC-SIGN and DC-SIGNR, respectively (*R*^2^ ≥ 0.99 for all fits). The intercept between the data set maxima (dotted line) and the linear fits for each protein were used to obtain the “apparent” PQRs required to achieve saturated QR binding (6.2 ± 0.3 and 32.5 ± 0.4 for DC-SIGN and DC-SIGNR, respectively).

In order to more empirically establish the binding modes between the two lectins and QR-DiMan, the effect of titrating protein against a fixed concentration of QR was analyzed using FRET and hydrodynamic size analysis. Binding of both lectins with QR-DiMan yielded an initial linear increase in the FRET ratio with an increasing PQR before reaching saturation (Figure 3). This behavior is similar to that observed previously for DC SIGN binding with QD-DiMan but is very different from the binding of DC-SIGNR with QD-DiMan, which displays a sigmoidal relationship with very little binding occurring at the low PQRs.^21^ This difference agrees with the aforementioned observation that a significant amount of strong affinity, higher order multivalency complexes are established for QR-DiMan·DC-SIGNR complexation, allowing significant binding to occur even at these lower PQRs. By fitting the linear region of the FRET ratio–PQR relationship and taking the intersection with the maximum recorded FRET ratio (Figure 3C), the “apparent” PQRs required to achieve saturated QR binding (i.e., maximal FRET ratio) are estimated as ∼6 for DC-SIGN and ∼33 for DC-SIGNR. Please note that these values do not represent the actual number of lectins that are bound to each QR-DiMan but, rather, the number of lectins per QR required to achieve saturate binding because not all added lectins will be able to bind to the QR under the natural association/dissociation equilibrium. As the overall binding affinity of QR-DiMan with DC-SIGNR is significantly weaker than that with DC-SIGN, the proportion of added DC-SIGNR molecules that are bound to the QR would be considerably lower than that for DC-SIGN. In addition, it is also worth noting that these values are likely to be smaller than the “true” PQR required to achieve saturated protein coverage of QR. This is because, according to our previous energy-dispersive X-ray spectroscopy mapping, the CdSe fluorescent core is situated at the center of the QR.^53^ Thus, only lectins bound within proximity of the central region will be close enough to engage in FRET due to the inverse sixth power dependency of the FRET efficiency to the distance between the donor and acceptor.^58^

**Figure 4:**
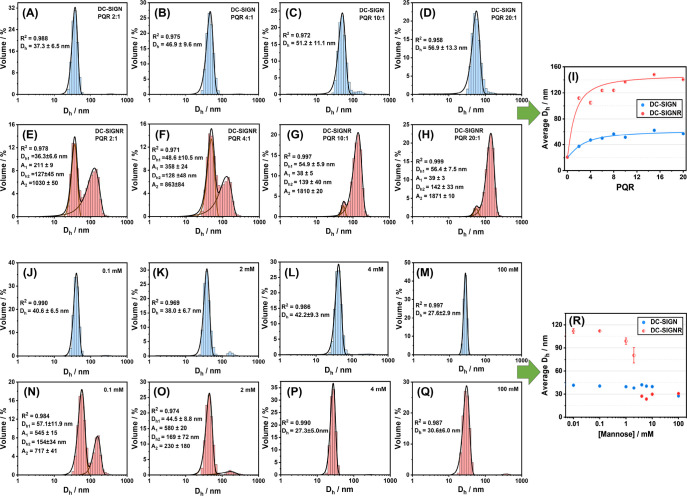
*D*_h_ distribution histograms of 10 nM QR-DiMan after binding with DC-SIGN at PQRs of (A) 2:1, (B) 4:1, (C) 10:1, and (D) 20:1 or binding with DC-SIGNR at PQRs of (E) 2:1, (F) 4:1, (G) 10:1, and (H) 20:1; a pre-incubated mixture of 10 nM QR-DiMan with 40 nM DC-SIGN after addition of free mannose at concentrations of (J) 0.1, (K) 2, (L) 4, or (M) 100 mM; and a pre-incubated mixture of 10 nM QR-DiMan with 40 nM DC-SIGNR after addition of free mannose at concentrations of (N) 0.1, (O) 2, (P) 4, and (Q) 100 mM. Data were fitted with Gaussian function, and the *D*_h_ values are given as mean ± 1/2 FWHM. (I) The corresponding average *D*_h_–PQR relationship (average *D*_h_ = *D*_h,1_ × *A*_1_ % + *D*_h,2_ × *A*_2_ % , where *A*_1_% and *A*_2_% are the percentage area of the Gaussian fits; filled circles: single distribution; half-filled circles: two distributions) fitted with an offset Hill function (*D*_h, PQR_ = *D*_h,0_ + (*D*_h, ∞_ – *D*_h,0_)/(1 + (PQR_50_/PQR)^*n*^) where *D*_h,0_ was fixed to the *D*_h_ of QR-DiMan, *D*_h, ∞_ = 62 ± 6 and 150 ± 10 nm, PQR_50_ (PQR at 50% of *D*_h, ∞_) = 2.7 ± 0.8 and 1.3 ± 1, and *n* = 1.3 ± 0.4 and 1 ± 1, for DC-SIGN and DC-SIGNR, respectively; *R*^2^ ≥ 0.99). (R) Plot of the average *D*_h_ against the free mannose concentration for QR-DiMan pre-incubated with DC-SIGN/R at PQR of 4:1. (DC-SIGN: blue; DC-SIGNR: red).

The binding modes of the two proteins with QR-DiMan were further probed by analyzing the hydrodynamic diameters (*D*_h_) of the resulting QR-lectin complexes. The apparent *D*_h_ values were obtained from Gaussian fits of the *D*_h_ distribution histograms over a PQR range of 0–20. For QR-DiMan·DC-SIGN complexation, only a single size distribution is observed, which plateaus at ∼60 nm after a PQR of ∼6 (Figure 4A–D,I). This value is approximately equal to the summation of the *D*_h_ values of a single QR-DiMan flanked by two proteins (where QR-DiMan and DC-SIGN demonstrate individual *D*_h_ values of ∼21 and ∼18 nm, respectively). This size is therefore likely to be representative of a monolayer of lectin with all CRDs simultaneously specifically bound to a single QR-DiMan particle. QR-DiMan·DC-SIGNR complexation, on the other hand, demonstrates two distinct size distributions (Figure 4E–H): one that plateaus at ∼60 nm, which matches well with that of DC-SIGN complexation, and another that plateaus at ∼140 nm. The similarity in *D*_h_ values between the smaller size peak in DC-SIGNR and that observed for DC-SIGN evidently confirms the presence of simultaneous binding in DC-SIGNR. Meanwhile, the larger size distribution with DC-SIGNR is indicative of the formation of cross-linked clusters, similar to that observed previously with QDs, although the size here is somewhat smaller.^21^ Fitting the average *D*_h_ (weighted with respect to their integrated areas) against PQR using an offset Hill function (see Figure 4I), it is observed that the average *D*_h_ plateaus at 62 ± 6 and 150 ± 10 nm for DC-SIGN and DC-SIGNR, respectively. This demonstrates that the number of DC-SIGNR clustered particles tends to increase with an increasing PQR. This result provides further evidence that the high affinity interaction observed at low concentrations for DC-SIGNR does indeed correlate with the simultaneous binding mode and the weaker affinity binding correlates with an increase in cross-linked binding at the higher PQRs. The fact that the weaker cross-linking binding mode appears to dominate, rather than being displaced, by the stronger simultaneous binding interaction at higher concentrations suggests that these two binding modes may occur at different regions of the QR.

**Figure 5:**
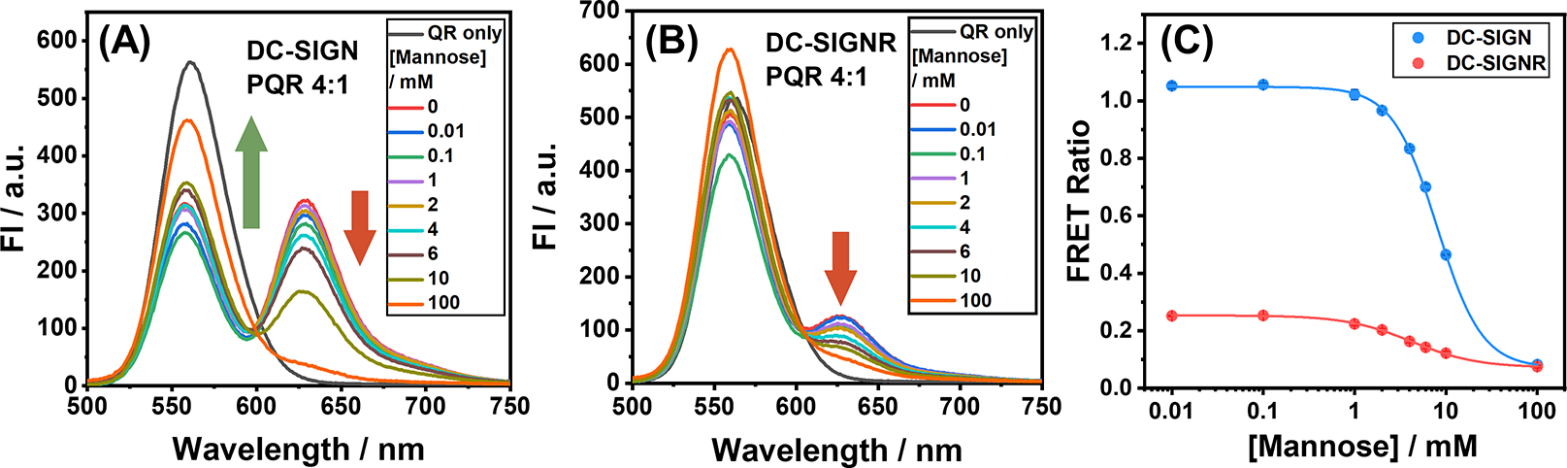
Direct excitation background corrected fluorescence spectra corresponding to increasing concentrations of free mannose ([Mannose]) to a pre-incubated 4:1 PQR mixture of 10 nM QR-DiMan with (A) DC-SIGN or (B) DC-SIGNR. (C) Plot of the relationship between the FRET ratio against the mannose concentration for the fluorescence spectra recorded in (A) and (B) fitted with an offset Hill function (eq 2; where *F*_0_ = 1.049 ± 0.007 and 0.253 ± 0.001, *F*_∞_ = 0.071 ± 0.003 and 0.072 ± 0.003, *K*_i_ (mannose concentration to give 50% inhibition) = 8.0 ± 0.1 and 4.2 ± 0.2 mM, and *n* = 1.78 ± 0.05 and 1.21 ± 0.08 for DC-SIGN (blue) and DC-SIGNR (red), respectively; *R*^2^ > 0.999 for both fits).

To confirm the binding affinity-mode relationship, free d-mannose was added to compete for pre-formed QR-DiMan·DC-SIGN/R complexes prepared under a PQR of 4:1. DLS analysis showed that the amount of clustered QR-DiMan·DC-SIGNR species was decreased even with addition of just 0.1 mM mannose (Figure 4N; Figure S11B). This is a clear indication that the cross-linked clusters correspond to the weaker binding mode, which is more easily displaced than the stronger simultaneous binding. The average *D*_h_ of the smaller species was also reduced to ∼30 nm with ≥4 mM mannose (Figure 4P), along with the complete disappearance of the clustered species, indicating the eventual breakdown of both binding modes. Moreover, addition of mannose to QR-DiMan·DC-SIGN/R complexes also led to a significant, dose-dependent reduction in FRET ratio (Figure 5), indicated by the simultaneous reduction of dye fluorescence and recovery of QR fluorescence. These results are fully consistent with free mannose-induced lectin·glycan-QR complex dissociation. The FRET ratio change with the mannose concentration ([Mannose]) was then fitted with an offset Hill equation to obtain the apparent-inhibition constant (*K*_i_), which represents the “apparent” concentration of mannose required to inhibit binding by 50% (eq 2; where *F* is the FRET ratio at a particular mannose concentration and *n* represents the Hill coefficient; Figure 5C; see Figure S6 for the normalized plot).
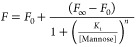
2Here, an FRET minimum of ∼0.07 is observed at high mannose concentrations for both proteins. This may result from a small amount of clustering induced by high amounts of mannose, observable by the hydrodynamic size at similar concentrations (i.e., ∼30 nm, see Figure 4M,Q, *vs* ∼20 nm for unbound QR-DiMan). The apparent-*K*_i_ values for DC-SIGN and DC-SIGNR were estimated as 8.0 ± 0.1 and 4.2 ± 0.2 mM, respectively. The higher *K*_i_ value for DC-SIGN is a reflection of its stronger overall binding affinity with QR-DiMan and hence requires a higher concentration of mannose in order to displace 50% of binding interactions. However, DC-SIGNR displays a broader decay with increasing mannose concentrations than DC-SIGN does, evidenced by the smaller exponent (*n* = 1.78 ± 0.05 *vs* 1.21 ± 0.08 for DC-SIGN *vs* DC-SIGNR). This suggests that mannose is able to displace a larger amount of bound DC-SIGNR at much lower concentrations than DC-SIGN. It is worth noting that, where the difference in the overall apparent-*K*_d_ between DC-SIGN/R binding to QR-DiMan is ∼100-fold, the difference in the apparent-*K*_i_ with mannose is only ∼2 fold. This may suggest that, for a PQR of 4:1, the simultaneous binding mode of DC-SIGNR provides a higher contribution to the apparent-*K*_i_ than the crosslinking mode, where the latter contributes mainly to broadening of the decay. Interestingly, because the fluorescent core of the QR has been shown to be located in the center of the cylindrical middle section of the QR,^53^ any binding at the middle section will have a larger influence on the apparent *K*_i_. This result may therefore suggest that the glycan display at the central cylindrical section of the quantum nanorod is better suited to form simultaneous binding with DC-SIGNR than cross-linking interactions. Consistent with this, the decay in *D*_h_ for the simultaneously bound complexes also occurs at the same mannose concentration range (Figure 4R). Given the previously established cross-linking character of spherical QD-DiMan with DC-SIGNR,^21^ this conclusion appears reasonable as any deviation in binding mode induced by QRs with a similar radius and chemical composition is expected to occur at the central cylindrical section, where the glycan display differs the most from that of QDs.

Overall, these results collectively demonstrate that our QR-DiMan probe is able to distinguish between the strong simultaneous and weak cross-linking binding modes by combining both FRET and hydrodynamic size analyses and that these binding modes are likely to favor different regions of the quantum nanorod.

### Binding Mode Rationale *via* S/TEM Imaging

**Figure 6:**
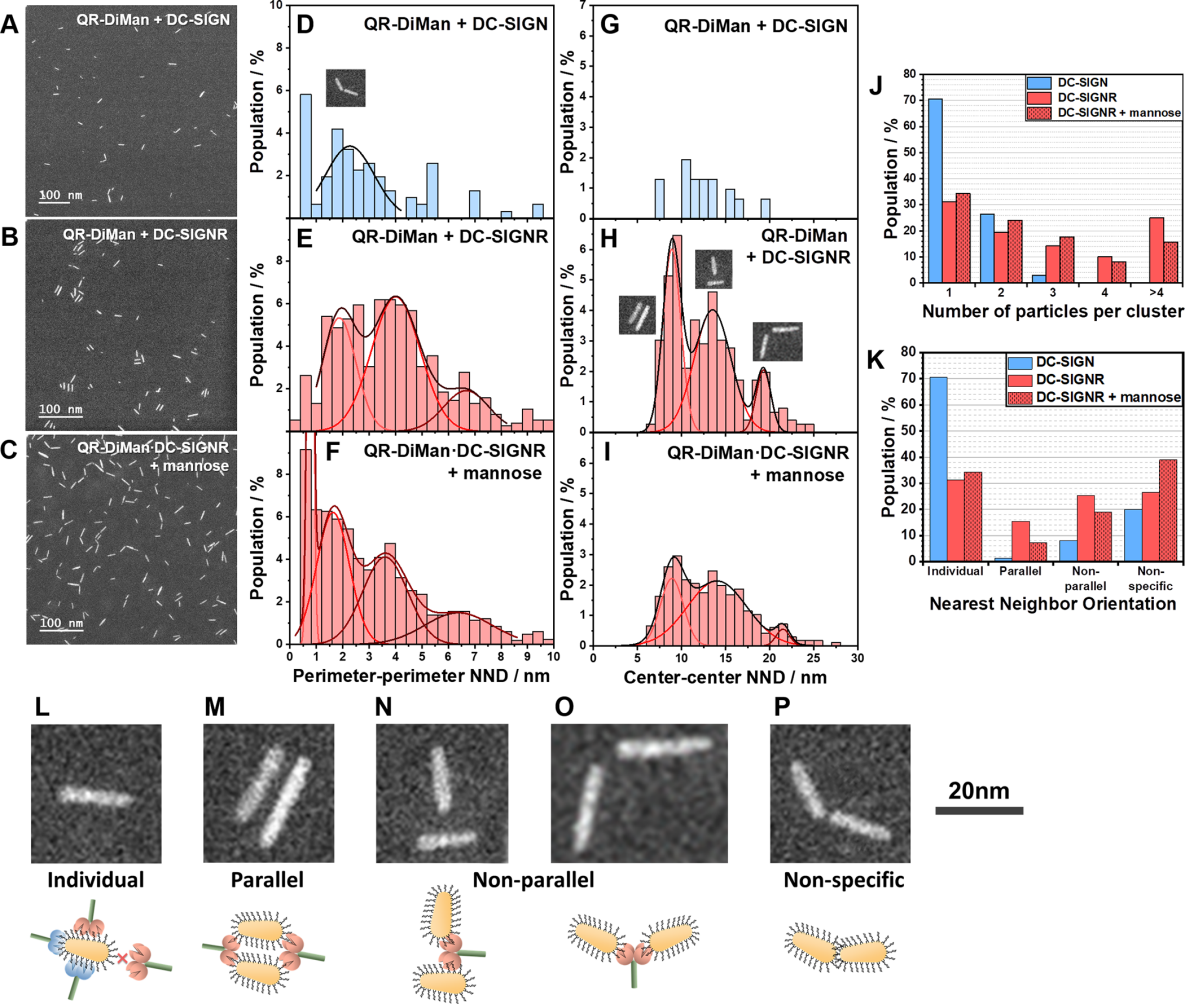
S/TEM images of cryo-prepared QR-DiMan (10 nM) after complexation with 4 molar equivalent of (A) DC-SIGN, (B) DC-SIGNR, and (C) DC-SIGNR in the presence of 2 mM mannose. Histograms of the ppNND, fitted with uni- or multimodal Gaussian fits, for QR-DiMan incubated with (D) DC-SIGN or (E) DC-SIGNR or (F) pre-incubated QR-DiMan·DC-SIGNR with 2 mM mannose. Histograms of the center–center nearest neighbor distances (ccNNDs, for 2.9 < ppNND < 7.7 nm), fitted with trimodal Gaussian fits (where applicable), for QR-DiMan incubated with (G) DC-SIGN or (H) DC-SIGNR or (I) pre-incubated QR-DiMan·DC-SIGNR with 2 mM mannose. Statistical analysis of the (J) numbers of QRs per cluster (i.e., the number of interconnected QRs with a ppNND of <7.7 nm) and (K) QR nearest neighbor orientations (individual: ppNND > 7.7 nm; parallel: the area of the first ccNND peak (or ccNND < 10 nm for DC-SIGN); non-parallel: the sum of the second and third ccNND peak areas (or ccNND > 10 nm for DC-SIGN); non-specific: ppNND < 2.9 nm). Schematics and example S/TEM images of (L) simultaneous binding of DC-SIGN (blue) to all sections of QR or DC-SIGNR (red) to the cylindrical section only; DC-SIGNR cross-linking QR-DiMan into (M) parallel stacks, (N) non-parallel perpendicular stacked QRs and (O) non-parallel adjacently stacked QRs; and (P) non-specifically adsorbed QRs (scale bar = 20 nm).

Electron microscopy was further employed to capture “snapshot” images of the QR-lectin complexes in order to provide a more detailed understanding of their interactions. Here, QR-lectin samples, prepared with a PQR of 4:1, were plunge-frozen and then vacuum dried before being placed for S/TEM imaging. We have shown previously that this method allows for the successful capture of the native dispersion state of nanoparticle assemblies.^21,59^ Binding of DC-SIGN demonstrated mostly isolated individual QRs (Figure 6A), which correlated nicely with the single *D*_h_ distribution for DC-SIGN·QR-DiMan complexes observed by DLS (Figure 4B). Additionally, binding of DC-SIGNR yielded both clustered QR assemblies and non-clustered individual QRs (Figure 6B), which again agreed well with the two distinct *D*_h_ species observed in DLS (Figure 4F).

To further quantify assembly formation, the inter-QR distances were analyzed by measuring the perimeter–perimeter nearest neighbor distances (ppNNDs) between each QR (i.e., the shortest distance between the perimeter of one QR with that of its nearest neighboring QR). The distributions of ppNNDs were then plotted as histograms and fitted with Gaussian distribution curves. This analysis revealed three well-defined species for QR-DiMan·DC-SIGNR assemblies with ppNNDs of 1.9 ± 0.8, 4.0 ± 1.1, and 6.7 ± 1.0 nm (mean ± 1/2 FWHM; Figure 6E; Table 2). DC-SIGN, on the other hand, displayed only one well-defined ppNND distribution at 2.3 ± 1.1 nm (Figure 6D; Table 2). These results allowed us to draw three conclusions. (1) The smallest ppNND species (∼2 nm) were observed in similar amounts for both DC-SIGN and DC-SIGNR (20–27%). Such distances are comparable to the thickness of the glycan ligand coating; thus, these QR assemblies are deemed to result from non-specific interactions (depicted in Figure 6P). (2) The species with ppNNDs ≳ 7.7 nm were found in 71% of QR-DiMan·DC-SIGN and 31% of QR-DiMan·DC-SIGNR complexes and were randomly distributed (Figure S15). These corresponded nicely to single QR-DiMan particles bound with a layer of protein, as observed by DLS, thus confirming that both DC-SIGN and DC-SIGNR were able to bind tetravalently with all CRDs with one QR-DiMan. (3) The well-defined species for DC-SIGNR binding with ppNNDs of 4.0 ± 1.1 and 6.7 ± 1.0 nm were not observed in significant amount with DC-SIGN (Figure 6D); such distances were consistent with the discrete distances expected for DC-SIGNR-bridged QRs. In addition, of the QRs with ppNNDs below ∼7 nm, 49% contained >2 QRs per cluster for DC-SIGNR, whereas such assemblies were only observable in negligible amounts for DC-SIGN (<3% of QRs). These multi-QR assemblies are consistent with the larger *D*_h_ sizes observed by DLS. Interestingly, these are only limited to a few QRs per cluster and thus do not resemble the extensive inter-cross-linked networks observed for QD-DiMan bridged by DC-SIGNR.^21^ This is likely due to the dual simultaneous and cross-linking binding mode, which imparts a limit to the number of cross-linking interactions possible.

**Table 2:** Summary of Fitting Parameters Obtained from the Gaussian Fits of the Nearest Neighbor Distances (NND) of QR-DiMan Particles Binding with DC-SIGN, DC-SIGNR, or DC-SIGNR with 2 mM Mannose where ppNND is the Perimeter–Perimeter NND and ccNND is the Center–Center NND where 2.9 < ppNND < 7.7 nm

	perimeter–perimeter NND				center–center NND			
protein	mean ppNND (nm)	FWHM (nm)	area (%)	R^2^	mean ccNND (nm)	FWHM (nm)	area (%)	R^2^
DC-SIGN	2.3 ± 0.1	2.2 ± 0.4	8 ± 1	0.747				
DC-SIGNR	1.9 ± 0.2	1.5 ± 0.5	8 ± 4	0.869	8.9 ± 0.1	2.4 ± 0.3	15 ± 2	0.939
	4.0 ± 0.2	2.2 ± 0.9	15 ± 6		13.5 ± 0.3	4.9 ± 0.8	21 ± 3	
	6.7 ± 0.7	2 ± 1	4 ± 3		19.3 ± 0.3	2.0 ± 0.6	4 ± 1	
DC-SIGNR + mannose	1.63 ± 0.09	1.5 ± 0.4	10 ± 3	0.987^a^	8.9 ± 0.1	3.1 ± 0.4	7 ± 2	0.980
	3.6 ± 0.2	2.0 ± 0.6	9 ± 3		14.0 ± 0.4	7.9 ± 0.9	18 ± 2	
	6.4 ± 0.6	3 ± 1	5 ± 2		21.5 ± 0.3	1.8 ± 0.6	1.1 ± 0.4	

a An additional peak was observed at ∼0.8 nm, which was an artifact of the image resolution and fit poorly to the data.

To investigate how mannose competes with QR-DiMan binding with DC-SIGNR, S/TEM images were performed on a sample of a pre-incubated QR-DiMan· DC-SIGNR complex with 2 mM mannose (Figure 6C,F). Here, an ∼40% reduction of clusters containing >4 QRs was observed (Figure 6J), consistent with the significantly reduced mean *D*_h_ observed by DLS (Figure 4O). In parallel to the decrease in cross-linking, an ∼ 10% increase in the proportion of individual complexes and an ∼50% increase in the proportion of non-specific interactions were observed (Figure 6K). This result may explain why the *D*_h_ of QR-DiMan did not completely return to its original size after dissociation of bound proteins observed at high mannose concentrations.

Though QR-FRET and hydrodynamic size analysis were able to suggest that the different binding modes of DC-SIGNR may favor specific regions of the QR, S/TEM analysis provides a direct visual representation of how QR-DiMan particles orient themselves with respect to each other in the presence of lectin, which can be used to infer regional details regarding cross-linking. Here, inter-QR nearest neighbor orientations were analyzed by measuring the center–center nearest neighbor distance (ccNND; the shortest distance between the center of one QR with that of its nearest neighbor) for all QRs with a ppNND between 4.0 ± 1.1 and 6.7 ± 1.0 nm. As expected, no clear ccNND distribution was observed for QR-DiMan·DC-SIGN complexes (Figure 6G). In sharp contrast, for QR-DiMan·DC-SIGNR complexes, three discrete distributions were obtained with Gaussian fits with ccNNDs of 8.9 ± 1.2, 13.5 ± 2.4, and 19.3 ± 1.0 nm (mean ± 1/2 FWHM; Figure 6H; Table 2), respectively. These distributions are representative of QRs, which are stacked either parallel (i.e., center to center; Figure 6M), perpendicular (i.e., end to center; Figure 6N), or adjacent (i.e., end to end; Figure 6O) to one another, respectively.

If cross-linking did not discriminate between the different regions on the QR, then, based on surface area alone, the most common inter-QR orientation would be QRs stacked loosely parallel to one another. However, the ccNND distributions (Figure 6K) show that this is not case. Instead, only 15% of QRs are stacked parallel to their nearest neighbor, while 25% of QRs display non-parallel nearest neighbor orientations. This result suggests that cross-linking favors the spherical ends over the central cylindrical section of the QR. In addition, the parallel interactions were the only nearest neighbor orientation that showed a significant reduction upon addition of the mannose competitor. Therefore, it is plausible that these parallel QR stacks are composed of protein cross-linking at both QR ends, which are then either fully dissociated into isolated particles or partially dissociated into non-parallel interactions in the presence of mannose. This is feasible as further cross-linking between adjacent QR-lectin complexes within the same original QR cluster would be much more kinetically favorable than cross-linking multiple QR-lectin complexes that are freely diffusing in the solution. Based on these results, together with those observed by FRET and hydrodynamic size analyses above, three interesting conclusions can be deduced: (1) DC-SIGNR favors cross-linking upon spherical geometries (e.g., QDs or the QR end sections), (2) DC-SIGNR favors simultaneous binding upon cylindrical geometries (e.g., the central region of the QR), and (3) DC-SIGN favors simultaneous binding indiscriminately upon both spherical and cylindrical geometries.

**Figure 7:**
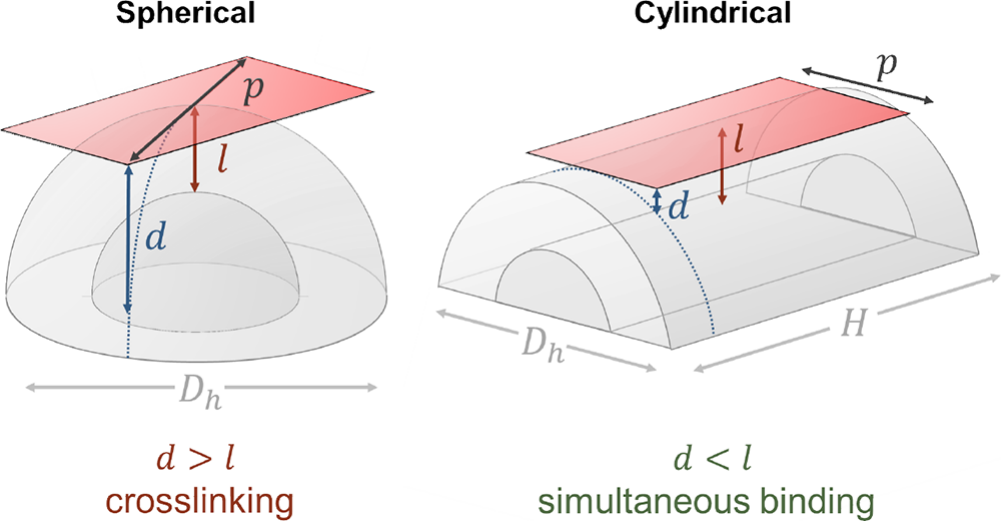
Schematic depicting the difference in separation distance between the binding contact area of DC-SIGNR (red) with either the spherical end (left) or cylindrical section (right) of the QR surface. Here, *d* is the separation distance between the protein contact area and the QR-DiMan surface, *l* is the maximum compression length of the surface glycan ligands, *p* is the Ca–Ca distance between two CRDs at the furthest separation distance from the QR surface, *D*_h_ is the estimated QR-DiMan hydrodynamic diameter, and *H* is the height of the QR cylindrical section. (Not to scale).

These different binding phenomena can be rationalized by considering the relative dimensions of both binding partners. The hydrodynamic dimensions of QR-DiMan can be estimated from the summation of the average QR core dimensions obtained by S/TEM (core length, *L*_core_^QR^ 14.7 ± 5.7 nm; core diameter, *D*_core_^QR^ 3.1 ± 0.7 nm; Figure S14) and the estimated hydrodynamic surface ligand length (2.9 ± 1.1 nm; derived from the previous QD-DiMan dimensions, where *D*_h_^QD^ = 9.5 ± 0.1 nm and *D*_core_^QD^= 3.7 ± 2.1 nm).^21^ This provided a QR-DiMan estimated terminal end *D*_h_ and cylindrical height (*H*) of 8.9 ± 2.3 and 11.6 ± 5.7 nm, respectively (see SI, Figures S14 and S16). Both of these dimensions are comparable with that of the equivalent QD-DiMan (*D*_h_^QD^ = 9.5 ± 0.1 nm).^21^ Due to the flexible nature of the EG_11_ chain, the glycan surface density and inter-glycan distance of each glycan ligand can be assumed to be roughly the same across the whole QR. Based on these geometrical values and a surface glycan valency of ∼370 per QR (see SI, Section 7), a glycan surface density of ∼0.63 glycans per nm^2^ and an average inter-glycan distance of ∼1.4 nm are obtained for QR-DiMan. The crystal structure of DC-SIGNR C-terminal tetrameric fragment (PDB code: 1XAR) provides approximate dimensions of 3.8 × 8.0 nm between the primary Ca^2+^ ions associated with glycan binding (see SI, Figure S17A).^60^ Thus, the binding contact area of each DC-SIGNR is likely to be smaller than both the spherical and cylindrical regions of the QR-DiMan. It would cover a surface area containing ∼20 glycans on QR-DiMan. These simple calculations suggest that neither the QR size nor the glycan surface density is likely to be the main factors causing the distinct binding modes for DC-SIGNR between the QR end and middle sections. Instead, the distinct DC-SIGNR binding modes are most likely to be determined by the large differences in the 3-dimensional surface curvatures between these regions. In fact, at the spherical ends of the QR, surface curvature could theoretically impart separation distances (*d*) as much as ∼4 nm from the protein binding sites to the glycan surface (eq 3) where *p* is the Ca–Ca distance between two CRDs at the furthest separation distance from the scaffold surface, which is taken as the average diagonal Ca–Ca distance (*p*:∼ 8.8 nm; Figure 7 left).
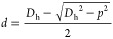
3

The theoretical maximum length at which the glycan ligands may be able to be compressed (*l*) can be derived from the estimated length of flexibility in the glycan ligand, i.e., *l* ∼2.1 nm for DHLA-EG_11_-DiMan (see SI, Section 7). Therefore, even with compression of the ligands, it would be impossible for all four binding sites to reach the glycan surface simultaneously. This means that the glycan displays at the QR ends are incapable of bridging all four binding sites in DC-SIGNR; thus, the protein cannot access its most stable simultaneously bound state. Instead, it is forced to find its next most favorable configuration by cross-linking with other particles to maximize binding enthalpy.^57^ For the cylindrical section of the scaffold, the curvature of the round of the cylinder is the same as the spherical ends; however, along the length of the cylinder, it is roughly flat. This means that the tetrameric lectin has the opportunity to align itself with its longer length parallel to the length of the quantum rod (Figure 7, right). Therefore, only the short length of the tetramer (i.e., *p* = 3.8 nm) needs to contribute to the separation distance (*d*) from the quantum rod surface, resulting in a *d* of only ∼0.4 nm. This is well within the maximum compression length of the ligands, therefore allowing glycans to easily bridge all four CRDs to give strong simultaneous tetravalent binding. Finally, the tetrameric model of DC-SIGN has been predicted to exhibit a more compact shape with an average diagonal inter-binding site distance of 5.6 nm (Figure S17B).^61^ This results in a much smaller separation distance between the protein contact area (*d* ≈ 1 nm), which is smaller than the maximum compression length of the flexible surface ligands (*l* = 2.1 nm). This would therefore easily allow the QR surface glycans to bridge all 4 CRDs, regardless of the region of the QR that DC-SIGN binds to, leading to the exclusively simultaneous binding mode as observed here. These calculations therefore show that while the tetrameric structures of both lectins are still unknown, and the good agreement between the predicted and observed results suggests that the structural models for DC-SIGN/R are likely to be relatively reliable. Moreover, it also demonstrates that, by taking into account nanoscaffold curvature, estimating the *d* – *l* relationship between the protein and nanoscaffold dimensions can provide a useful prediction of the binding mode for multimeric lectins.

## Conclusions

In summary, we have presented a new glycan-quantum rod-based multifunctional biophysical probe for multivalent lectin-glycan interactions. By combining FRET, hydrodynamic size, and S/TEM imaging analysis, we have dissected the geometric influences of glyco-nanomaterials on the MLGI properties of DC-SIGN and DC-SIGNR, a pair of tetrameric lectin models with almost identical monovalent binding motifs but distinct binding site arrangements. We demonstrate that, given an ample polyvalent glycan density and area, nanoscale scaffold curvature has a fundamental impact on the binding modes of their MLGIs. Here, DC-SIGNR is able to distinguish between the end and middle sections of QR-DiMan, forming strong simultaneous tetravalent binding at the central cylindrical section but bis-bivalent cross-linking at the spherical ends. Meanwhile, DC-SIGN binds with strong simultaneous tetravalent binding irrespective of the QR section. We have further predicted that only curvatures affording a separation distance between the protein contact area and the glycan ligand surface (*d*) smaller than the compression length of the glycan ligands (*l*) can result in the strong simultaneous binding of all binding sites. However, if *d* is greater than *l*, then only weak cross-linking or other lower valency binding interactions can occur.

This glycan-QR probe thus provides a powerful new tool for studying the shape-dependent binding affinities and mechanisms of multivalent lectins at the molecular level. It can provide a fundamental rationale behind the MLGI shape dependency. Such information can act as a useful guidance on the shape design of multivalent glycans for targeting specific MLGIs in a solution. These results also demonstrate how multimeric lectins like DC-SIGN/R differentiate glycan displays with different geometries, which may help explain some of their differences in virus-binding and transmitting properties in cells. Future studies will investigate the shape selectivity of such lectin receptors with glycan-coated QDs and QRs at cell surfaces to reveal the difference and correlation between MLGIs occurring in solutions and on cell surfaces. In addition, other design elements such as scaffold size, ligand density, and flexibility will also be investigated in order to design optimal glycan nanoparticles for potent and specific targeting of MLGIs in the body for therapeutic interventions.

## Experimental Section

### Materials

CdSe/CdS-elongated (dot-in-a-rod) core/shell quantum rods (QR) capped with mixed TOPO/TOP/HPA surface ligands in hexane were purchased from Centre for Applied Nanotechnology GmbH (Germany). H_2_O used was ultra-pure (resistance: >18.2 MΩ·cm) purified by an ELGA Purelab classic UVF system. The binding buffer consisted of 20 mM HEPES, 100 mM NaCl, 10 mM CaCl_2_, pH 7.8, in H_2_O. All other chemicals and reagents were purchased commercially and used as received unless stated otherwise. DHLA-EG_11_-DiMan were synthesized in-house using our previously established protocols.^21^ MS: calculated *m*/*z* for C_60_H_111_N_5_O_27_S_2_ (DHLA-EG_11_-DiMan) [M + 2H]^2+^: 699.84, found: 699.95.

### Preparation of QR-DiMan^21,22^

QR-DiMan were prepared using our established quantum dot cap exchange protocol. Briefly, QR (416 μL, 2 nmol) in hexane was precipitated by EtOH (12 mL) and spun at 15,000*g* for 5 min. After discarding the clear supernatant, the resulting QR pellet was dissolved in CHCl_3_ (300 μL) and then a mixture of DHLA-EG_11_-DiMan (273 μL, 5.4 μmol) in CHCl_3_ and NaOH (64 μL, 6.48 μmol) in EtOH was added. MeOH (100 μL) was further added, and the resulting mixture was covered with foil and stirred at r.t. for 30 min. The resulting QRs were then precipitated by addition of hexane (600 μL) and spun at 15,000*g* for 3 min. The resulting QR pellet was dissolved in H_2_O (300 μL) and washed with H_2_O (3×200 μL) using a 30 kDa MWCO spin filter. This yielded a stable water-dispersed QR-DiMan (1.55 nmol) in 78% yield. The supernatants and washes were combined, freeze-dried, and then used to determine the unbound ligand *via* the sulfuric acid-phenol carbohydrate quantification (SI, Section 2.2). The ligand amount bound to the QR was calculated from the difference between that added and that remaining unbound post cap-exchange,^57^ yielding a mean glycan surface coverage of 370 ± 30 ligands per QR.

### FRET Assays

All FRET studies were measured using a Cary Eclipse Fluorescence Spectrophotometer in a 0.7 mL quartz cuvette (optical path length: 10 mm). Samples were prepared by adding protein to QR-DiMan in the binding buffer containing BSA (1 mg/mL, to minimize non-specific interactions and adsorptions on surfaces) and incubated for 20 min before measurement. Samples were excited with λ_EX_ = 450 nm (corresponding to the absorption minimum of Atto-594 to minimize dye direct excitation background), and the fluorescence spectra were recorded from 500 to 750 nm, with intervals (Δλ) of 1 nm. Mannose competition experiments were performed in the same way, where after addition of d-mannose, the resulting solution was incubated for a further 20 min before measurement. All fluorescence spectra were corrected by the background fluorescence of the same concentration of labeled lectin, without QR, under identical conditions. The PMT voltages and instrument slit widths were adjusted to avoid signal saturation at high concentrations. While varying instrument setups will affect the absolute fluorescence signals of both the QR and dye, it will not affect the FRET ratio used in data analysis due to its ratiometric character.^21,57^

### Hydrodynamic Size Analysis

All hydrodynamic size studies were performed on a Malvern Zetasizer Nano using 10 mm PMMA cuvettes.^20^ Samples were prepared by adding protein to QR-DiMan in the binding buffer and incubating for 20 min before measurement. The mannose competition experiment was performed the same way by adding d-mannose to pre-incubated QR-lectin samples and further incubation for 20 min. Distributions were obtained by averaging a minimum of three measurements of 10 runs of 10 s. Mean hydrodynamic diameters (*D*_h_) were obtained by fitting these averaged volume hydrodynamic size distribution histograms with either unimodal or bimodal Gaussian distribution functions.^20^

### S/TEM Imaging^21,57^

All S/TEM images were taken using an FEI Titan^3^ Themis 300 G2 S/TEM. Samples were prepared under the same conditions as those used for *D*_h_ analysis. A small sample mixture (3.5 μL) was loaded onto a plasma-cleaned TEM grid with a continuous carbon support film before being blotted and then plunge-frozen into liquid ethane. The TEM grids were then allowed to warm up to room temperature over several minutes by placing the specimens in a liquid nitrogen-cooled storage container in a rotary-pumped vacuum desiccator. The samples were plasma-cleaned (15 s) and then analyzed using high-angle annular dark field scanning transmission electron microscopy mode. S/TEM Images were analyzed by using ImageJ 1.4.3.67 software to obtain perimeter–perimeter (pp) and center–center (cc) nearest neighbor distance (NND) histograms. The mean NNDs were obtained by fitting the distributions with multimodal Gaussian distribution curves.

### Data Analysis and Fitting

All fluorescence data were analyzed using Microsoft Excel 2016. The FRET ratio data were presented as mean ± standard errors of two repeats at each concentration. The FRET ratio–concentration plots were fitted by the Origin software (version 2019b) using the relevant equations, taking into account the SEs of each experimental data point, to give the best fits (highest *R*^2^ values). The results from the best fits were then listed in the relevant tables and displayed as mean ± the standard fitting errors.

## Supporting Information Available

Materials, instruments, and methods; preparation and characterization of QR-DiMan; characterization of QR-DiMan lectin hydrodynamic sizes; STEM image analysis; and curvature analysis (PDF)

## Abbreviations

CRD: carbohydrate recognition domain
DC-SIGN: Dendritic Cell-Specific Intercellular adhesion molecule-3-Grabbing Nonintegrin
DC-SIGNR: a DC-SIGN related lectin found on endothelial cells
*D*_h_: hydrodynamic diameter
DHLA-EG_11_-DiMan: dihydrolipoic acid-undeca(ethylene glycol)-α-1,2-manno-biose
FRET: Förster resonance energy transfer
HRMS: high resolution mass spectrometry
*K*_d_: apparent equilibrium binding dissociation constant
MLGI: multivalent lectin-glycan interactions
PQR: protein: QR molar ratio
QD: quantum dot
QR: quantum nanorod
S/TEM: scanning/ transition electron microscopy
