# Supplementary material for: Polyvalent Glycan Functionalized Quantum Nanorods as Mechanistic Probes for Shape-Selective Multivalent Lectin-Glycan Recognition
Source: ACS Appl Nano Mater. 2023 Mar 14;6(6):4201–13. doi: 10.1021/acsanm.2c05247 (PMC10043877; doi:10.1021/acsanm.2c05247)
Supplement: Supplementary file 1 — an2c05247_si_001.pdf [file an2c05247_si_001.pdf]

Supporting Information

Polyvalent Glycan Functionalized Quantum Nanorods as Mechanistic Probes for Shape-Selective Multivalent Lectin-Glycan Recognition

James Hooper,[1] Darshita Budhadev,[2] Dario Luis Fernandez Ainaga,[3] Nicole Hondow,[3] Dejian Zhou,[2]* and Yuan Guo[1]*

[1] School of Food Science & Nutrition and Astbury Centre for Structural Molecular Biology, University of Leeds, Leeds LS2 9JT, United Kingdom.

[2] School of Chemistry and Astbury Centre for Structural Molecular Biology, University of Leeds, Leeds LS2 9JT, United Kingdom.

[3] School of Chemical and Process Engineering, University of Leeds, Leeds LS2 9JT, United Kingdom.

Email: d.zhou@leeds.ac.uk (D.Z.) or y.guo@leeds.ac.uk (Y.G.)

1 Materials, Methods and Instrumentation

1.1 Materials

Phenol (>99.5%) was purchased from Alfa Aesar. D-mannose was purchased from Biosynth Carbosynth. Bovine serum albumin (BSA, >99%) was purchased from BioServUK. CdSe/CdS elongated core/shell quantum rods (QR_{560}; core diameter = 3.1 ± 0.7 nm, core length = 15 ± 6 nm based on our TEM images; 1st excitonic λ_{abs} = 541 nm, λ_{em} = 558 nm, nominal fluorescence quantum yield = 68 %) capped with mixed ligands of trioctylphosphine oxide, trioctylphosphine and hexadecylamine, in hexane, was purchased from Centre for Applied Nanotechnology (CAN) GmbH (Germany). 2-[4-(2-hydroxyethyl) piperazin-1-yl]ethanesulfonic acid (HEPES, >99%); 2-amino-2-(hydroxymethyl)propane-1,3-diol (tris base, >99.8%); calcium chloride ($CaCl_2$, fused granular); chloroform ($CHCl_3$, >99.8%); ethylenediamine tetraacetic acid, disodium salt dehydrate (ETDA, >99%); hydrochloric acid (HCl, ~37%); sodium chloride (NaCl, >99.5%); sodium hydroxide (NaOH, pellets, >99%) and sulfuric acid (H_2SO_4, >95%) were purchased from Fischer Scientific. Hexane (>97%) was purchased from Sigma-Aldrich. Ethanol absolute (EtOH, >99.97%) was purchased from VWR Chemicals BDH®. H_2O used was ultra-pure (resistance >18.2 MΩ.cm), purified by an ELGA Purelab classic UVF system.

Glycans and linkers were synthesized in-house and purified using our previously established protocols.[1,2] DHLA-EG_{11}-DiMan was synthesized by a copper-free "click" reaction between LA-EG_{11}-Cyclooctyne and N_3-EG_2-DiMan followed by reduction using TCEP.HCl as reported previously.[2,3] MS: calculated *m/z* for $C_{60}H_{111}N_5O_{27}S_2$ (DHLA-EG_{11}-DiMan) $[M+H]^{2+}$ 699.84, found 699.95.

All buffers were made up using ultrapure water. These include: loading buffer, 25 mM Tris pH 7.8, 1.25 M NaCl, 25 mM $CaCl_2$; binding buffer, 20 mM HEPES pH 7.8, 100 mM NaCl, 10 mM $CaCl_2$; elution buffer, 20 mM HEPES pH 7.8, 100 mM NaCl, 2.5 mM EDTA; binding buffer for protein labeling, 20 mM HEPES pH 7.2, 100 mM NaCl, 10 mM $CaCl_2$; and elution buffer for labeled proteins, 20 mM HEPES pH 7.2, 100 mM NaCl, 2.5 mM EDTA.

1.2 Methods and Instrumentation

Centrifugation was performed using either a Thermo Scientific Haraeus Fresco 17, Heraeus Multifuge 3SR or a Beckman Coulter Avanti JXN-30 centrifuge, depending on the speed and volume, at room temperature (r.t.)

unless otherwise stated. Concentration or washing by centrifugation was carried out using Sartorius Stedim Lab 30 kDa molecular weight cut-off (MWCO) Vivaspin 500 and Merck Millipore 10 kDa MWCO Amicon Ultra centrifugal filters for QRs and protein, respectively. Dialysis was performed using Thermofischer Scientific 14kDa MWCO BioDesign Dialysis Tubing. Evaporation was performed using at reduced pressure using Genevac Concentrator EZ-2 or a Virtis Benchtop K freeze dryer.

High resolution mass spectrometry (HRMS) was used to analyze all protein samples and was performed using a Bruker Daltonics MicroTOF mass spectrometer. Deconvoluted mass values reported are in Da and protein labeling efficiency was obtained from the ratio of the integral of the labelled protein HRMS peak to the sum of that of the labeled and unlabeled protein peaks. Liquid chromatography mass spectroscopy (LC-MS) was used to analyze all other samples and was performed using a Bruker AmaZon speed mass spectrometer. Ultraviolet-visible light spectroscopy (UV-vis) was performed on either a Cary 60 UV-vis spectrophotometer using an Agilent Technologies sub-micro 10 mm quartz cell or a Thermo Scientific Nanodrop 2000 spectrophotometer with optical path length of 1 mm using a droplet of sample.

2 QR-Glycan Preparation and Characterization

2.1 QR-DiMan General Characterization

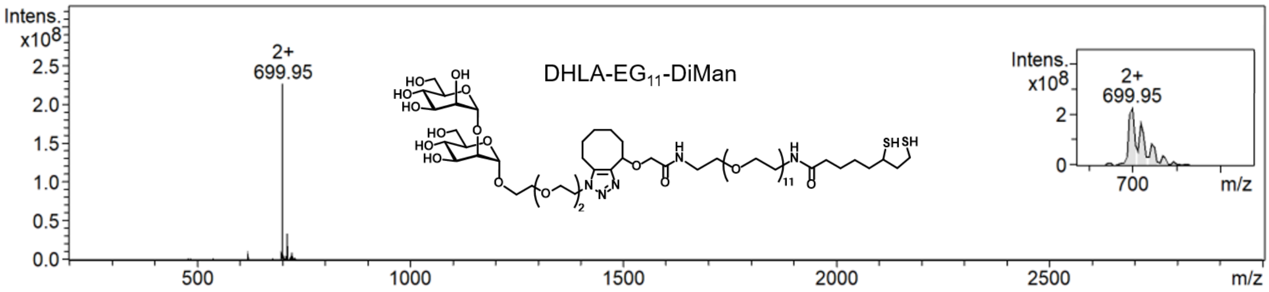

Figure S1. LC-MS of DHLA-EG$_{11}$-DiMan (expected $[M+H]^{2+}$ 699.84, found 699.95).

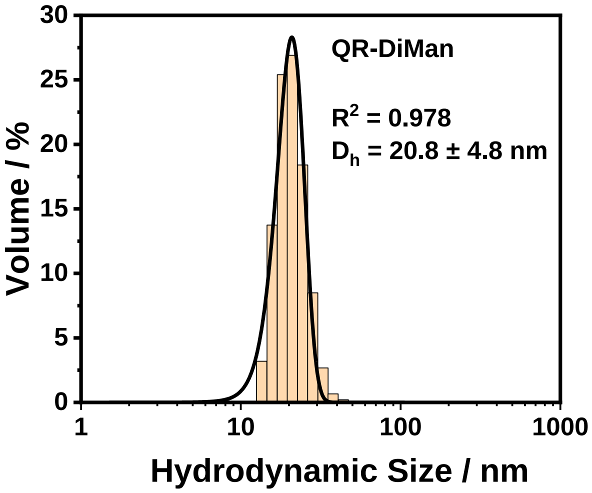

Figure S2. D_h distribution histogram of QR$_{560}$ capped with LA-EG$_{11}$-DiMan (QR-DiMan) measured by DLS, fitted with a log-normal Gaussian distribution function.

2.2 QR Surface Glycan Ligand Valency Determination

The average number of ligands conjugated to the QR surface was determined using the sulfuric acid-phenol method of carbohydrate quantification.[5] Calibration was performed using the N_3-EG_2-DiMan precursor, as described previously,[6] to obtain an extinction coefficient at 490 nm of 18900 ± 200 $M^{-1}cm^{-1}$. Samples were obtained by freeze drying the combined supernatants and washes obtained from the QR change exchange with DHLA-EG_{11}-DiMan and dissolving in H_2O (1.6 mL). Phenol (5 % w/w in H_2O, 80 µL) and sulfuric acid (400 µL) were then simultaneously added to a diluted solution of sample (5 µL) and H_2O (75 µL), before being vortexed and left at r.t. for 30 min. Average absorbance were then measured using UV-vis spectroscopy to obtain the concentration and, consequently, amount of unconjugated ligand using the calibration curve below (A_{490} = 0.508 ± 0.004, [Glycan] = 26.9 ± 0.2 µM, n_{unconj} = 4.82 ± 0.04 µmol, respectively). This was then subtracted from the amount of ligand added to obtain the amount of conjugated ligand, and divided by the moles of QR (n_{added} = 5.4 µmol and n_{QR} = 1.55 nmol of QR-DiMan, respectively) to provide an average surface coverage of 370 ± 30 DHLA-EG_{11}-DiMan ligands per QR.

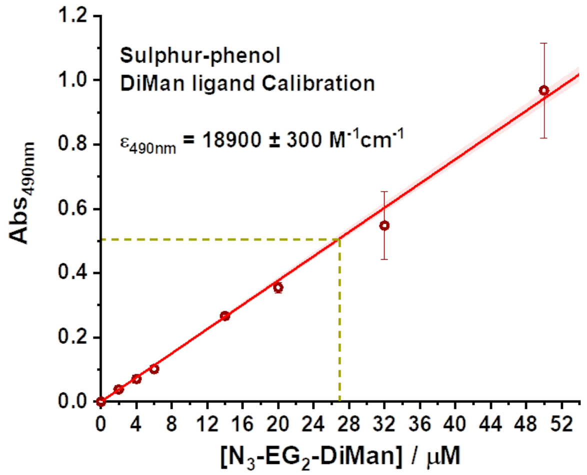

Figure S3. Calibration curve of absorbance at 490 nm vs. the concentration of the N_3-EG_2-DiMan standard used for glycan valency determination (slope = 18900 ± 200 M^{-1}; R^2 = 0.999).

2.3 QR-Atto594 Spectral Overlap and Förster Radius

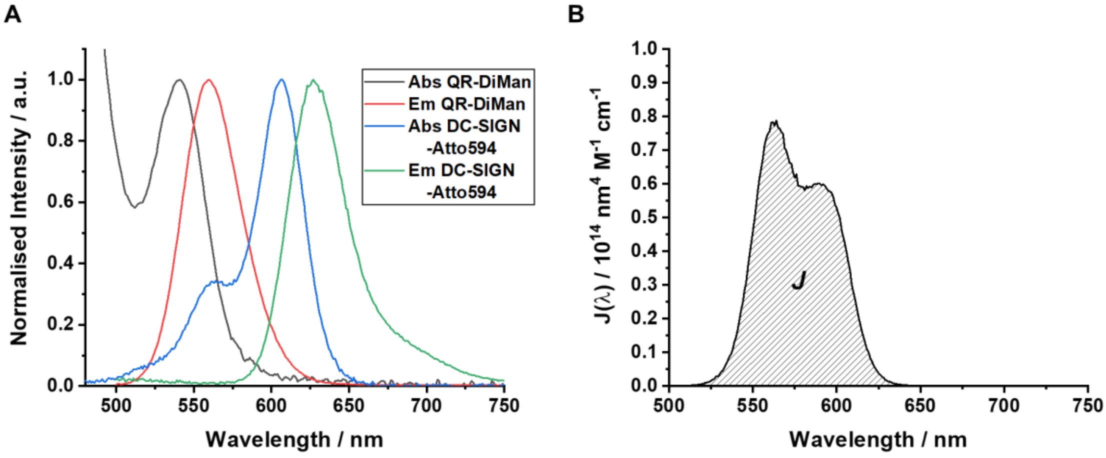

Figure S4. (A) Normalized absorption and emission spectra of QR-DiMan (black and red, respectively) and DC-SIGN-Atto594 (blue and green, respectively); and (B) the spectral overlap, $J(\lambda)$, of QR-DiMan (donor) emission with DC-SIGN-Atto594 (acceptor) absorbance, obtained by Equation S1.

The spectral overlap, $J(\lambda)$, was calculated by Equation S1, where $I_D(\lambda)$ is the normalized donor fluorescence intensity as a function of wavelength and $\varepsilon_A(\lambda)$ is the extinction coefficient of the acceptor as a function of wavelength (Atto-594: $\varepsilon_A(\mathrm{max}) = 1.2 \times 10^5\ \mathrm{M^{-1}\,cm^{-1}}$). The integral of the spectral overlap is obtained to be $J = 4.22 \times 10^{15}\ \mathrm{nm^4\,M^{-1}\,cm^{-1}}$.

$$J = \int J(\lambda)\ \mathrm{d}\lambda = \int \frac{I_D(\lambda)\varepsilon_A(\lambda)\lambda^4}{\int I_D(\lambda)\ \mathrm{d}\lambda}\ \mathrm{d}\lambda \qquad \text{S1}$$

The quantum yield (QY), Φ, of the QRs were obtained using Rhodamine 6G in ethanol (QY = 95%, λ_{ex} = 480 nm) as a reference. Here, the QY before and after conjugation with DHLA-EG$_{11}$-DiMan was found to be 49 % and 6.2 %, respectively, indicating an 8-fold reduction of QY after cap-exchange. This is likely caused by cap-exchange induced surface defects due to ligand etching of the outer protective shell of the QR surface.[7]

The Förster radius, R_0, was obtained using Equation S2, where κ^2 is the dipole orientation factor (κ^2 = 2/3, assuming randomly oriented dipoles) and n_r is the refractive index (n_r = 1.33 for the binding buffer).[4,8] This provided an R_0 value of 48 Å.

$$R_0 = \left(8.79 \times 10^{-5} \cdot \frac{\kappa^2 \cdot \Phi_D \cdot J}{{n_r}^4}\right)^{\frac{1}{6}} \qquad \text{S2}$$

3 Production, Labelling and Characterization of Protein

All proteins were derived from plasmids, made in-house, containing the wild-type extracellular amino acid identities of DC-SIGN and DC-SIGNR which have previously been shown to retain their tetrameric structures and mannose binding capability.[3] Plasmids encoding for the DC-SIGN Q274C and DC-SIGNR R287C were made in-house via site directed mutagenesis, as described previously.[3] Amino acid identities of both proteins used are shown below, where the positions of the cysteine site mutation are denoted in bold and identical residues are indicated by an asterisks.

```
 DC-SIGN: KVPSSISQEQSRQDAIYQNLTQLKAAVGELSEKSKLQEIYQELTQLKAAVGELPEKSKLQEIYQELTRLKAA
DC-SIGNR: KVPSSLSQEQSEQDAIYQNLTQLKAAVGELSEKSKLQEIYQELTQLKAAVGELPEKSKLQEIYQELTRLKAA
          ***** ***** ************************************************************

 DC-SIGN: VGELPEKSKLQEIYQELTWLKAAVGELPEKSKMQEIYQELTRLKAAVGELPEKSKQQEIYQELTRLKAAVGEL
DC-SIGNR: VGELPEKSKLQEIYQELTRLKAAVGELPEKSKLQEIYQELTRLKAAVGELPEKSKLQEIYQELTELKAAVGEL
          ****************** ************* ********************** ******** ********

 DC-SIGN: PEKSKQQEIYQELTRLKAAVGELPEKSKQQEIYQELTQLKAAVERLCHPCPWEWTFFQGNCYFMSNSQRNWHD
DC-SIGNR: PEKSKLQEIYQELTQLKAAVGELPDQSKQQQIYQELTDLKTAFERLCRHCPKDWTFFQGNCYFMSNSQRNWHD
          ***** ******** *********  **** ****** ** * ***** **  ********************

 DC-SIGN: SITACKEVGAQLVVIKSAEEQNFLQLQSSRSNRFTWMGLSDLNQEGTWQWVDGSPLLPSFKQYWNRGEPNNVG
DC-SIGNR: SVTACQEVRAQLVVIKTAEEQNFLQLQTSRSNRFSWMGLSDLNQEGTWQWVDGSPLSPSFQRYWNSGEPNNSG
          * *** ** ******* ********** ****** ******************** ***  *** ***** *

 DC-SIGN: EEDCAEFSGNGWNDDKCNLAKFWICKKSAASCSRDEEQFLSPAPATPNPPPA
DC-SIGNR: NEDCAEFSGSGWNDNRCDVDNYWICKKPAA-CFRDE
           ******** ****  *     ***** ** *  **
```

(Bold in the original: the Q in "...FMSNSQRNWHD" of DC-SIGN and the R in "...FMSNSQRNWHD" of DC-SIGNR.)

All proteins were expressed in *E. Coli* and purified using mannose-sepharose affinity chromatography, as described previously.[3,6] DC-SIGN Q274C and DC-SIGNR R287C were labelled with maleimide-Atto594 and purified again to remove excess dye. Protein concentrations were obtained by UV-vis spectroscopy using Equation S3, where $x_{protein}$ is the monomeric protein concentration, A_λ is the absorbance at wavelength λ,

CF_{280} is the dye correction factor at 280 nm (Atto594: 0.50; no dye: 0.00), and ε_{280} is the protein extinction coefficient at 280 nm (DC-SIGN: 70400 M^{-1} cm^{-1}; DC-SIGNR: 60890 M^{-1} cm^{-1}).

$$x_{protein} = \frac{A_{280} - CF_{280} \cdot A_{607}}{\varepsilon_{280}} \quad \text{S3}$$

Protein molecular weight (M_W) and labelling efficiency (LE) were confirmed from deconvoluted HRMS data, as reported previously.[6] Calculated protein M_W values were obtained from the amino acid sequences of one monomeric unit of the corresponding protein and LE was obtained from the ratio of the labelled protein to unlabeled protein peak areas.

DC-SIGN: calculated M_W [DC-SIGN] 39197.22, found 39202.44.

DC-SIGN Q274C-Atto594: calculated M_W [DC-SIGN Q274C] 39172.22, [DC-SIGN Q274C + 2Ca] 39252.40, [DC-SIGN Q274C-Atto594] 40100.22; found 39253.60 and 40105.75. Labeling efficiency: 87%.

DC-SIGNR: calculated M_W [DC-SIGNR] 37478.99; found 37470.17.

DC-SIGNR R287C-Atto594: calculated M_W [DC-SIGNR R287] 37425.95, [DC-SIGNR R287C + 2Ca]: 37506.10, [DC-SIGNR R287C-Atto594] 38353.95; found: 37507.48 and 38358.26. labeling efficiency: 85%.

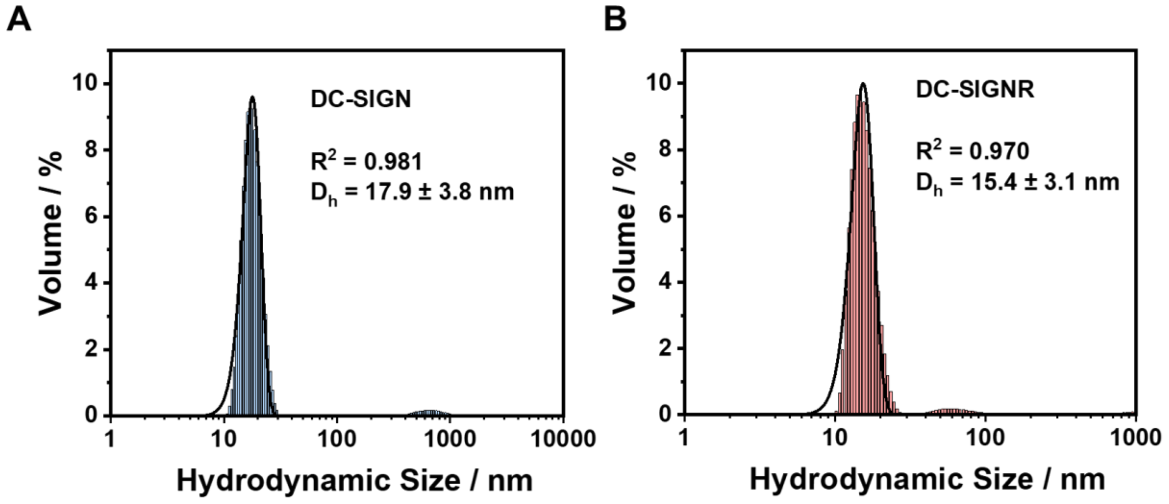

Figure S5. $\boldsymbol{D_h}$ **distributions histograms measured by DLS for (A) wild type DC-SIGN and (B) wild type DC-SIGNR. Fitted with log-normal Gaussian distribution functions.**

4 FRET Studies

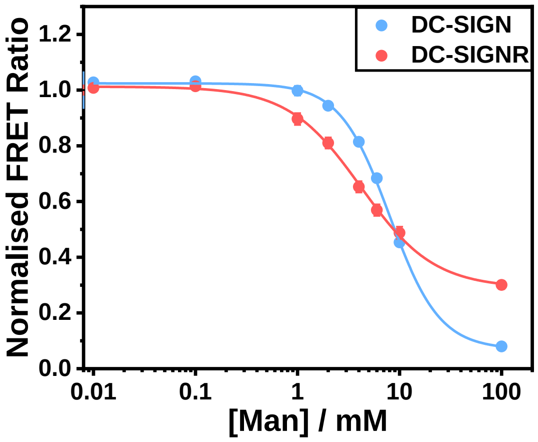

Figure S6. A plot of the normalized FRET ratio of pre-incubated QR-DiMan with DC-SIGN (blue) or DC-SIGNR (red) with increasing concentrations of mannose (i.e. manuscript Figure 5, normalized by the FRET ratio before mannose addition), fitted with an offset Hill function (see manuscript, eq. 2; where F_0 = 1.02 ± 0.06 and 1.01 ± 0.01, F_∞ = 0.069 ± 0.002 and 0.29 ± 0.03, K_i = 8.03 ± 0.09 and 4.2 ± 0.5 mM, and n = 1.78 ± 0.05 and 1.2 ± 0.2, for DC-SIGN and DC-SIGNR, respectively; $R^2 > 0.995$ for both fits). As both DC-SIGN and DC-SIGNR display a similar none-zero F_∞ value prior to normalization (manuscript, Figure 5). A much weaker initial FRET signal (*F₀*) for DC-SIGNR over DC-SIGN means the residual FRET accounts for a much higher proportion of the normalized FRET signal in DC-SIGNR than that in DC-SIGN, making such comparisons inadequate.

5 Hydrodynamic Size Studies

5.1 Lectin Titration Analysis via DLS

QR-DiMan (10 nM) was added with varying amount of wild-type protein at a protein: QR molar ratio (PQR) of 0 to 20. The samples were incubated in binding buffer at r.t. for 20 min before DLS measurement was performed and the results are shown below and fitted with Gaussian fits. From here, area weighted average D_h values were taken (average $D_\mathrm{h} = D_{\mathrm{h},1} \times A_1\% + D_{\mathrm{h},2} \times A_2\%$, where $A_1\%$ and $A_2\%$ are the percentage area of the Gaussian fits) and plotted against PQR. These were then fitted with an offset Hill equation in order to obtain the D_h at plateau.

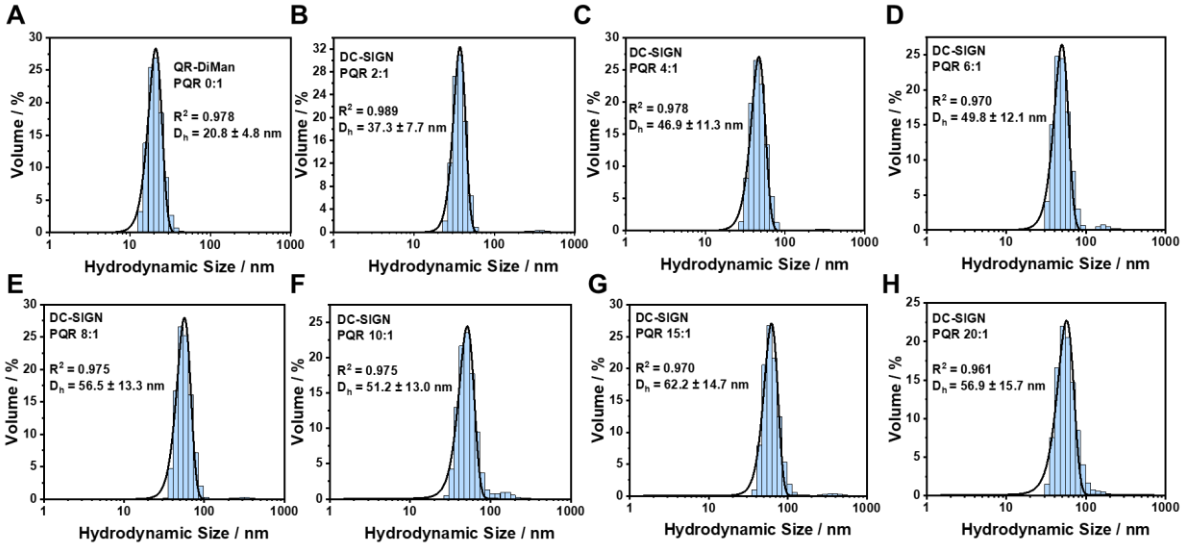

Figure S7. D_h distribution histograms fitted with Gaussian fits for the binding of QR-DiMan (10 nM) with DC-SIGN at varying protein: QR molar ratios (PQR) of (A) 0:1, (B) 2:1, (C) 4:1, (D) 6:1, (E) 8:1, (F) 10:1, (G) 15:1 and (H) 20:1.

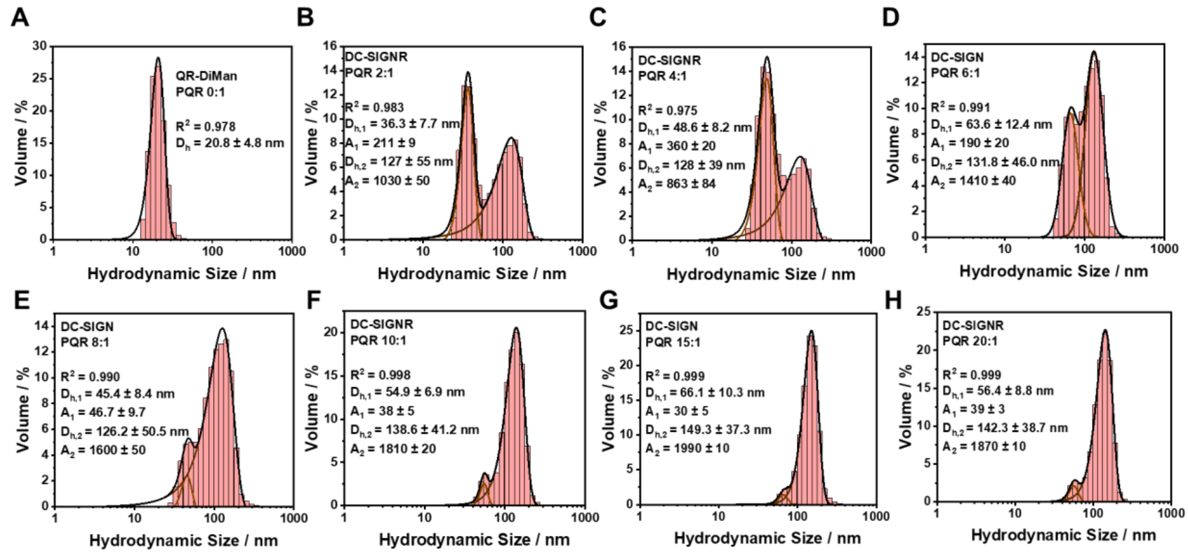

Figure S8. D_h distribution histograms fitted with multimodal Gaussian fits for the binding of QR-DiMan (10 nM) with DC-SIGNR at protein: QR molar ratios (PQR) of (A) 0:1, (B) 2:1, (C) 4:1, (D) 6:1, (E) 8:1, (F) 10:1, (G) 15:1 and (H) 20:1.

Table S1. Summary of parameters for the multimodal Gaussian fits of the D_h distribution histograms for the binding of QR-DiMan with DC-SIGN/R. Where PQR is the protein to QR molar ratio; $D_{h,i}$ is the mean hydrodynamic size; FWHM$_i$ is the full width at half maximum; and A_i is the integrated area. (i is the distribution mode number; all errors represent the standard deviation as observed by the fitting).

Lectin	PQR	$D_{h,1}$ / nm	FWHM$_1$ / nm	A_1	$D_{h,2}$ / nm	FWHM$_2$ / nm	A_2	R^2
	0	20.9 ± 0.2	9.7 ± 0.4	290 ± 10				0.978
DC-SIGN	2	37.3 ± 0.2	15.3 ± 0.4	530 ± 10				0.988
	4	46.9 ± 0.4	22.7 ± 0.9	660 ± 20				0.975
	6	49.8 ± 0.5	24 ± 1	680 ± 30				0.970
	8	56.5 ± 0.4	27 ± 1	790 ± 30				0.975
	10	51.2 ± 0.4	26 ± 1	680 ± 30				0.975
	15	62.2 ± 0.5	29 ± 1	840 ± 30				0.970
	20	56.9 ± 0.6	31 ± 1	750 ± 30				0.961
DC-SIGNR	2	36.3 ± 0.3	15.5 ± 0.6	211 ± 9	127 ± 2	111 ± 6	1030 ± 50	0.981
	4	48.6 ± 0.5	16 ± 2	360 ± 20	128 ± 3	79 ± 2	860 ± 80	0.991
	6	63.6 ± 0.7	25 ± 2	190 ± 20	132 ± 1	92 ± 3	1410 ± 40	0.991
	8	45 ± 1	17 ± 3	47 ± 10	126 ± 1	101 ± 3	1600 ± 50	0.987
	10	54.9 ± 0.8	14 ± 3	38 ± 5	138.7 ± 0.4	82 ± 1	1810 ± 20	0.998
	15	66 ± 1	21 ± 3	30 ± 5	149.3 ± 0.2	75 ± 1	1990 ± 10	0.999
	20	56.4 ± 0.7	18 ± 2	39 ± 3	142.3 ± 0.2	77 ± 1	1871 ± 10	0.999

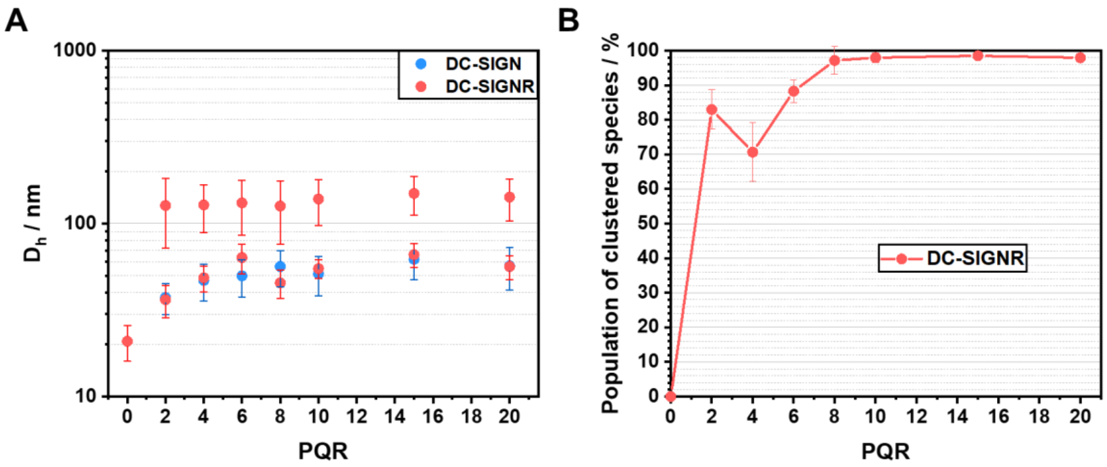

Figure S9. Plots the change in hydrodynamic size distribution, obtained by DLS, for the change in PQR for the binding of QR-DiMan with DC-SIGN (blue) or DC-SIGNR (red), measured by (A) the mean D_h ± ½FWHM, which demonstrates the similarity in the change in size of the smaller QR-DiMan·DC-SIGN/R complexes with increasing amount of protein, and (B) the percentage population of clustered species ($100\% \times A_2/(A_1 + A_2)$, where A_i is the integrated area of the ith mode of the multimodal Gaussian distribution fit), which demonstrates the increase in clustered species with protein concentration.

5.2 QR-Glycan-Lectin Dissociation with Mannose via DLS

Samples were prepared by adding wild-type protein (40 nM) to QR-DiMan (10 nM) in binding buffer and incubating at r.t. for 20 min. D-mannose was then added, with concentrations ranging from 0.01-100 mM, before incubating for a further 20 min and then measurement.

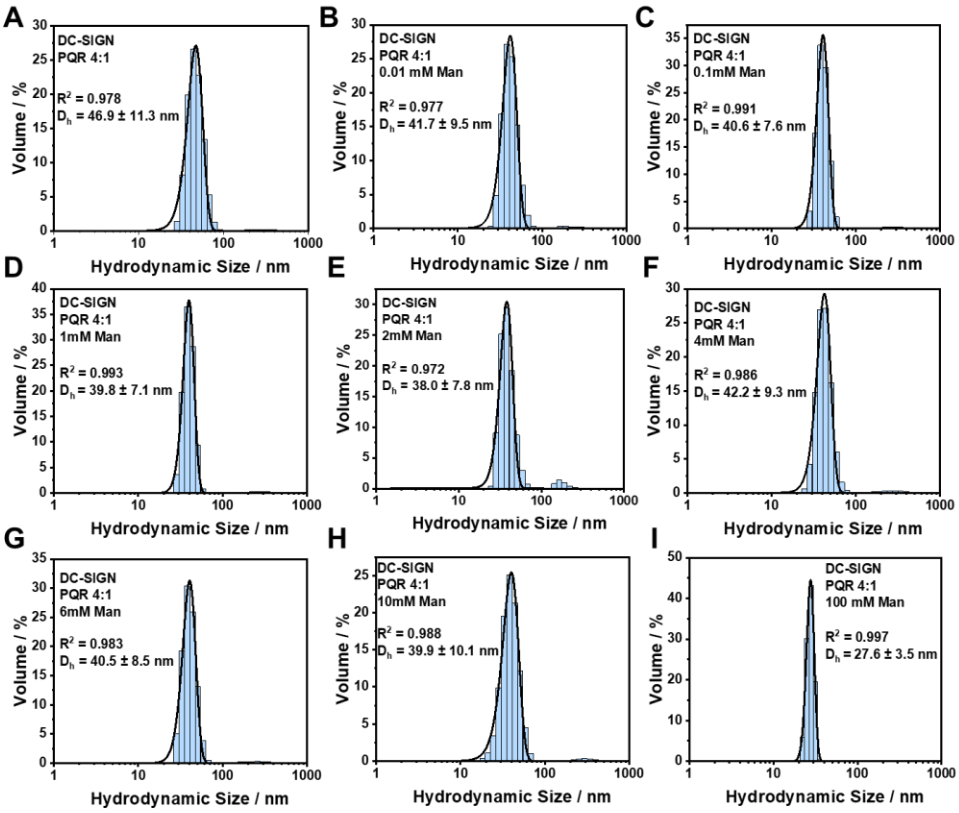

Figure S10. D_h distribution histograms for the pre-incubated QR-DiMan (10 nM) with DC-SIGN (PQR 4:1) after addition of (A) 0.01 mM, (B) 0.1 mM (C) 1 mM, (D) 2 mM, (E) 4 mM, (F) 6 mM, (G) 10 mM and (H) 100 mM of free mannose.

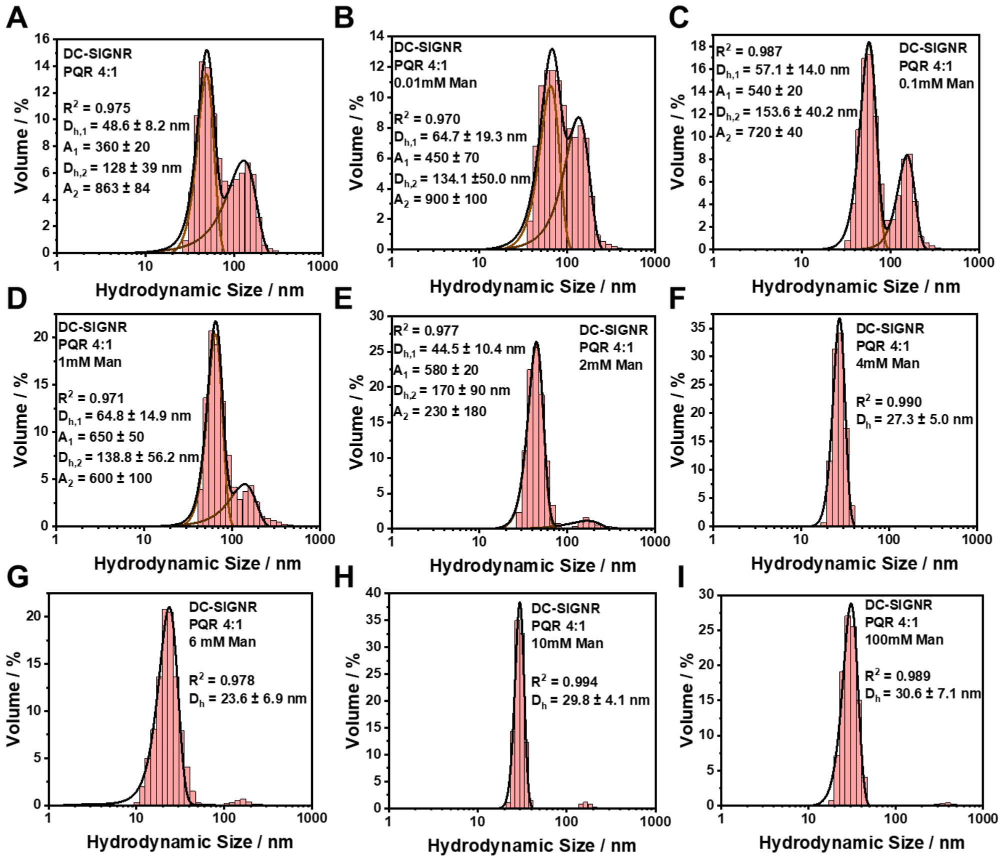

Figure S11. D_h distribution histograms for the pre-incubated QR-DiMan (10 nM) with DC-SIGNR (PQR 4:1) after addition of (A) 0.01 mM, (B) 0.1 mM (C) 1 mM, (D) 2 mM, (E) 4 mM, (F) 6 mM, (G) 10 mM and (H) 100 mM of free mannose.

Table S2. Summary of parameters for the multimodal Gaussian fits of the D_h distribution histograms for the dissociation of QR-DiMan·DC-SIGN/R with free D-mannose. Where [Man] is the concentration of D-mannose; $D_{h,i}$ is the mean hydrodynamic size; FWHMi is the full width at half maximum; and A_i is the integrated area (i is the distribution mode number; all errors represent the standard deviation as observed by the fitting).

Lectin	[Man] / mM	$D_{h,1}$ / nm	FWHM1 / nm	A_1	$D_{h,2}$ / nm	FWHM2 / nm	A_2	R^2
DC-SIGN	0	43.4 ± 0.2	16.7 ± 0.5	610 ± 20				0.985
	0.01	41.7 ± 0.3	19 ± 0.7	570 ± 20				0.976
	0.1	40.6 ± 0.1	15.2 ± 0.3	580 ± 10				0.99
	1	39.8 ± 0.1	14.1 ± 0.3	568 ± 10				0.993
	2	38 ± 0.3	15.7 ± 0.6	510 ± 20				0.971
	4	42.2 ± 0.2	18.6 ± 0.5	580 ± 10				0.985
	6	40.5 ± 0.2	17 ± 0.5	570 ± 20				0.982
	10	39.9 ± 0.2	20.3 ± 0.5	550 ± 10				0.988
	100	27.6 ± 0.1	6.9 ± 0.1	328 ± 4				0.997
DC-SIGNR	0	48.6 ± 0.5	25 ± 1	360 ± 20	128 ± 3	110 ± 10	860 ± 80	0.971
	0.01	65 ± 1	39 ± 3	450 ± 70	134 ± 5	100 ± 10	900 ± 100	0.965
	0.1	57.1 ± 0.3	28 ± 0.8	540 ± 10	154 ± 2	80 ± 5	720 ± 40	0.984
	1	64.8 ± 0.6	30 ± 2	650 ± 50	139 ± 9	110 ± 30	600 ± 100	0.966
	2	44.5 ± 0.3	20.8 ± 0.8	580 ± 20	170 ± 30	200 ± 100	200 ± 200	0.974
	4	27.3 ± 0.1	10 ± 0.3	390 ± 10				0.988
	6	23.6 ± 0.2	13.8 ± 0.6	310 ± 10				0.975
	10	29.8 ± 0.1	8.3 ± 0.2	339 ± 7				0.993
	100	30.6 ± 0.2	14.3 ± 0.4	440 ± 10				0.986

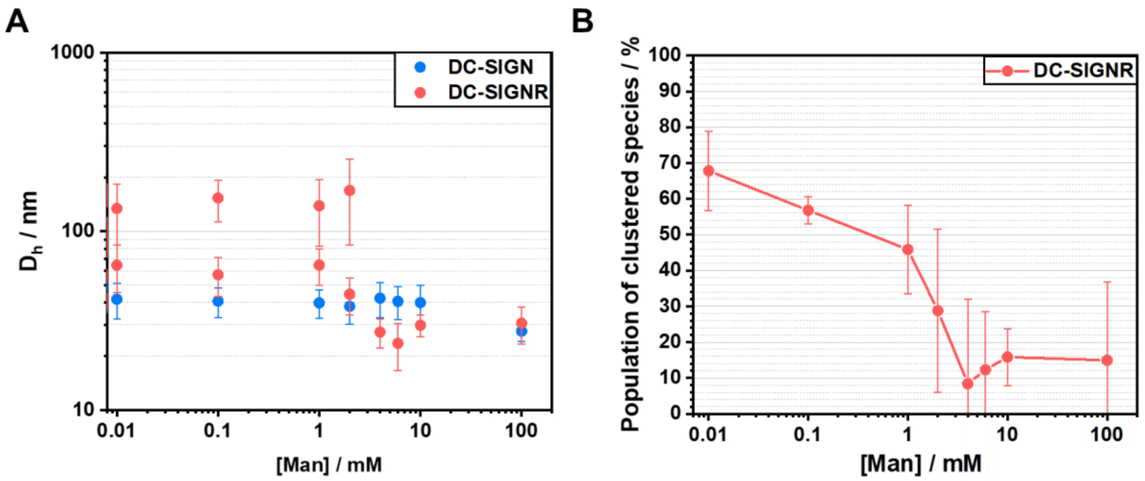

Figure S12. Plots the change in hydrodynamic size distribution, obtained by DLS, for the dissociation of a PQR 4:1 QR-DiMan·DC-SIGN (blue) or QR-DiMan·DC-SIGNR (red) complex with varying mannose concentration ([Man]), measured by (A) the mean D_h ± ½FWHM and (B) the percentage population of clustered species ($100\% \times A_2/(A_1 + A_2)$, where A_i is the integrated area of the ith mode of the multimodal Gaussian distribution fit). This demonstrates that the decrease in the population of clustered species for DC-SIGNR begins at lower [Man] than the decrease in the size of the smaller QR-DiMan·DC-SIGNR clusters, indicating that the lectin-glycan-QR interactions in the larger complexes are weaker in affinity than those of the smaller complexes.

6 STEM Studies

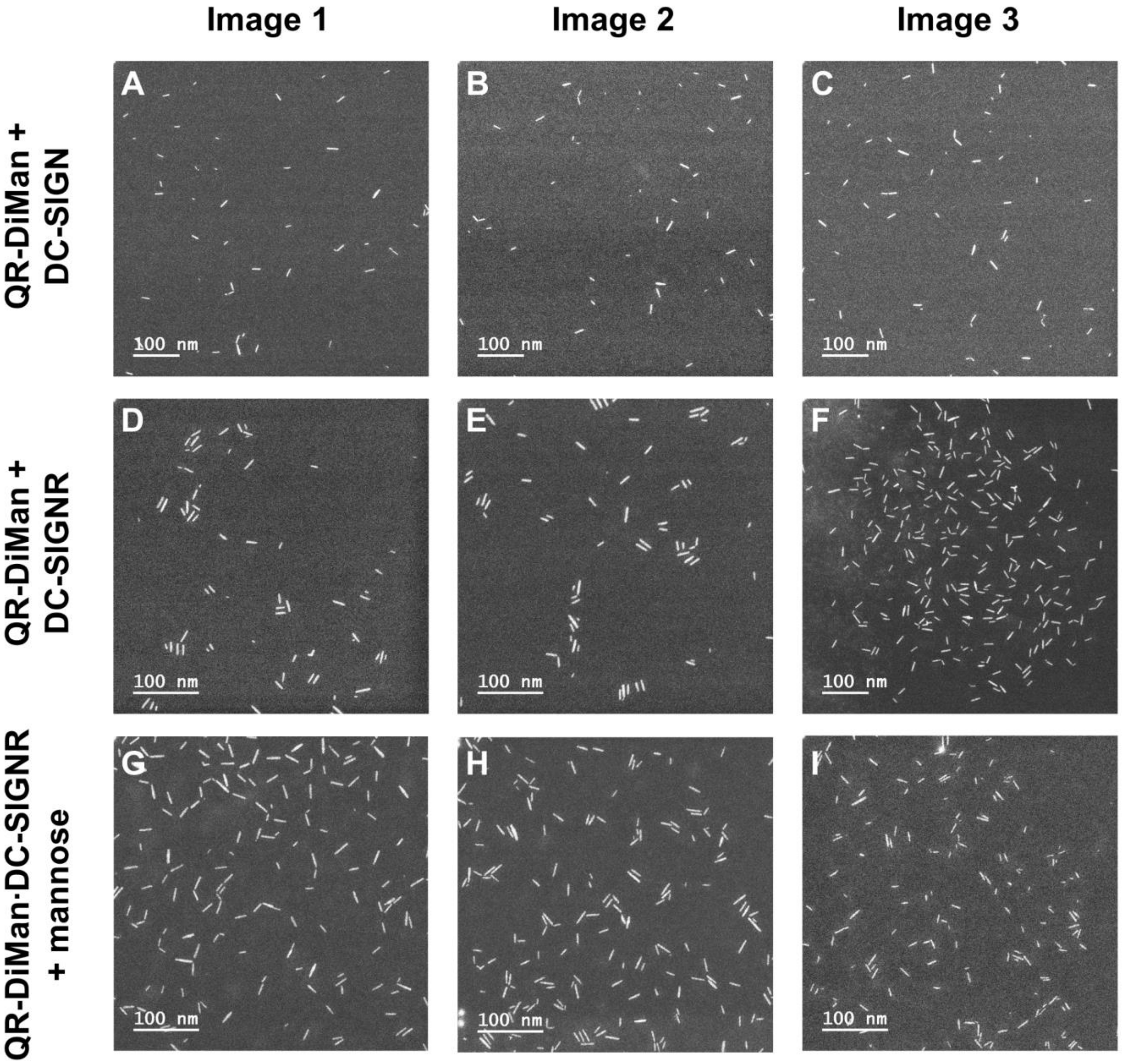

Figure S13. Typical high angle annular dark field scanning transmission electron microscope (HAADF STEM) images for PQR 4:1 samples of (A-C) QR-DiMan with DC-SIGN; (D-F) QR-DiMan with DC-SIGNR; and (G-I) a pre-incubated sample of QR-DiMan and DC-SIGNR with 2mM of D-mannose (Man). Each incubated for 20 min prior to plunge-freezing and subsequent imaging.

HAADF STEM images of each of the QR samples were analyzed by ImageJ software in order to obtain the nearest neighbor distances. Data was collected from 310 QRs from 12 images for QR-DiMan + DC-SIGN, 759 QRs from 15 images for QR-DiMan + DC-SIGNR and 1661 QRs from 15 images for QR-DiMan-DC-SIGNR + D-mannose, taking into account only particles within a threshold area of 5-500 nm^2.

Core diameter and length distributions were obtained from the area (a) and aspect ratios (AR) of each QR obtained by STEM imaging (where diameter, $d = \sqrt{a/\mathrm{AR}}$; length, $l = \sqrt{a \cdot \mathrm{AR}}$) and were plotted as log histograms and fitted with Gaussian fits to obtain a mean core diameter and length of D_{core} = 3.06 ± 0.70 nm and L_{core} = 14.7 ± 5.7 nm, respectively (Figure S14A). Interestingly, these values are ~64% smaller than the reported sizes prior to ligand conjugation which may suggest some etching of the QR surface upon cap

exchange. STEM images obtained from the analogous LA-EG_{11}-DiMan capped QDs (QD-EG_{11}-DiMan) imaged in Guo, *et al.,* 2017 were also analyzed for comparison (Figure S14B).[2]

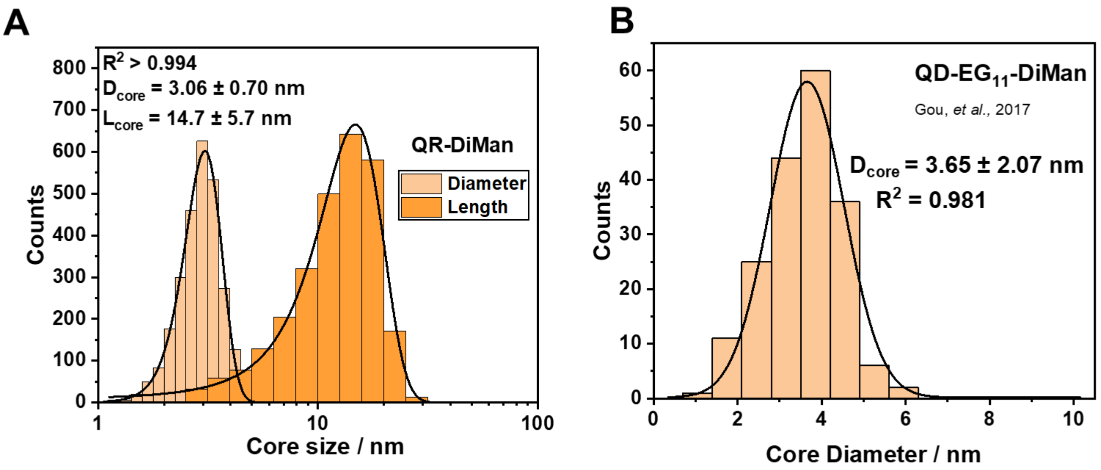

Figure S14. Histograms of (A) the QR diameter and length distributions obtained from STEM for the complexation of all images containing QR-DiMan; and (B) the diameter distribution obtained from the STEM images of QD-EG_{11}-DiMan reported in Guo, et al., 2017.[2] All plots fitted with Gaussian fits to obtain the mean core diameter and lengths.

Nearest neighbor distance (NND) distributions were obtained from the shortest inter-QR perimeter to perimeter or center-center distance (referred to as ccNND and ppNND, respectively) of each QR observed by STEM imaging. By plotting a matrix of ccNND against ppNND, it can easily be seen that a significant amount of QRs displayed randomly distributed isolated particles with ppNNDs of >10 nm (Figure S15A-C). DC-SIGNR, also displayed a significant amount of clustered particles with ppNNDs <10 nm, which were much fewer in number and more randomly dispersed for DC-SIGN. For DC-SIGNR, these smaller ppNND could be sub-grouped into high and low ccNND (<10 nm and >10 nm, respectively) which correspond to parallel stacked and non-parallel stacked inter-QR orientations. Both DC-SIGN and DC-SIGNR displayed a small number of very small inter-QR distances (i.e. <2 nm) which are likely too close to be bridged by a lectin binding partner and are thus deemed to be non-specific inter-QR interactions. In order to more statistically analyze the different inter-QR NNDs, ppNNDs distributions of <10 nm were fitted with multimodal Gaussian fits to obtain values and proportions of clustered, non-clustered and non-specific complexes. Those which fell within the range of the second and third distribution modes of DC-SIGNR (i.e. 4.0 ± 1.1 nm to 6.7 ± 1.0 nm) were deemed clustered complexes; those which fell under were deemed non-specific binding interactions; and those which were over were deemed non-clustered. Clustered QRs were further analyzed to obtain values for the nearest neighbor orientations by measuring the ccNND for QRs with ppNNDs between 4.0 ± 1.1 nm to 6.7 ± 1.0 nm. For DC-SIGNR, these distributions were fitted with Gaussian fits whereby the first, second and third peaks were representative of parallel, perpendicular and adjacent stacked inter-QR orientations, respectively. For DC-SIGN, parallel stacked rods were quantified as the proportion of QRs with ccNNDs <10 nm and non-parallel rods with ccNNDs >10 nm (where ppNND is between 4.0 ± 1.1 nm to 6.7 ± 1.0 nm). The number of QRs per cluster were quantified as the number of interconnected QRs with ppNNDs < 6.7 ± 1.0 nm.

.

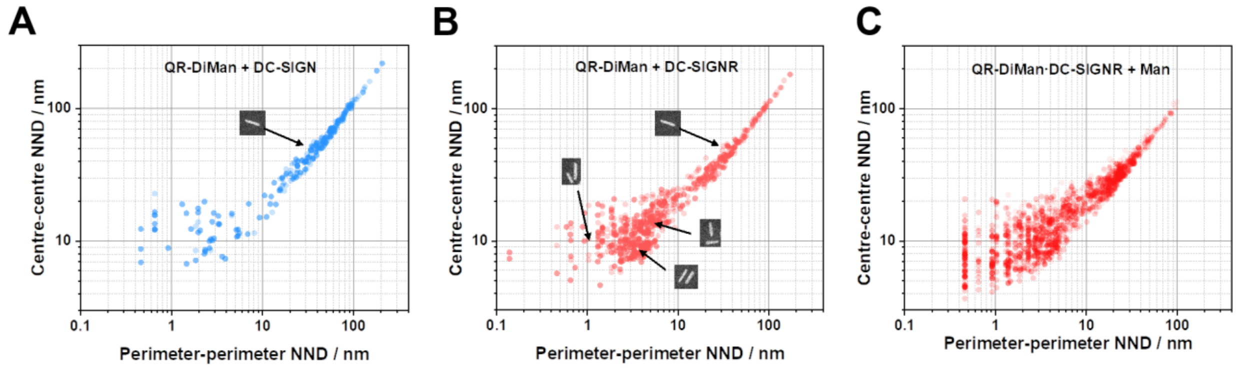

Figure S15. Comparison of inter-QR center to center NNDs (ccNND) to inter-QR perimeter to perimeter ppNNDs (NND) for each QR obtained from the STEM images of QR-DiMan incubated with (A) DC-SIGN, (B) DC-SIGNR or (C) a pre-incubated QR-DiMan·DC-SIGNR with D-mannose. Distributions demonstrate the regions associated with crosslinking (ppNND ~2-10 nm, in parallel and non-parallel inter-QR orientations, ccNND <10 nm and ccNND >10 nm, respectively), simultaneous binding (ppNND >10 nm) or non-specific QR adsorption (ppNND <2 nm).

7 Model of Glycan Display and Lectin Binding

Though exact values for hydrodynamic length (L_h) and diameter (D_h) cannot be obtained due to the limitations of DLS. Approximate values for these can be estimated by adding the average hydrodynamic length of the bound LA-EG$_{11}$-DiMan ligands ($l_{h,ligand}$) onto the known core dimensions of the QR. $l_{h,ligand}$ can be determined from the QD-EG$_{11}$-DiMan scaffolds described previously in Guo, *et al.*, 2017.[2] Here, the hydrodynamic size and core size were obtained to be 9.5 ± 0.1 nm and 3.65 ± 2.07 nm (see Figure S14B), obtained by DLS and STEM, respectively, which provides a $l_{h,ligand}$ for the surface LA-EG$_{11}$-DiMan ligands of 2.92 ± 0.05 nm (where, $l_{h,ligand} = ½(D_h - D_{core})$; Figure S16). The average core length and diameter of QR-DiMan were obtained by STEM to be 14.8 ± 5.7 nm and 3.06 ± 0.70 nm, respectively (Figure S14A). Thus, the average hydrodynamic length and diameters can be estimated to be around L_h = 20.6 ± 6.1 nm and D_h = 8.9 ± 2.1 nm, respectively.

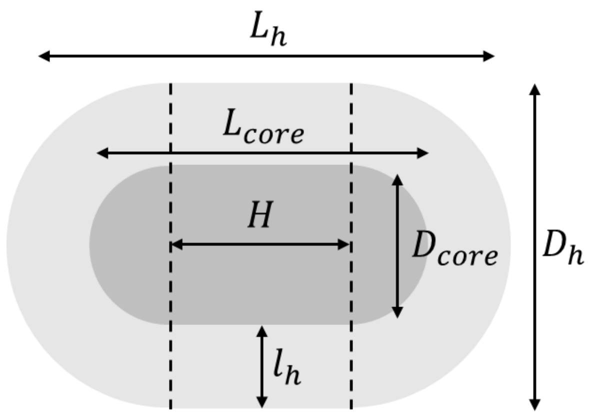

Figure S16. 2-dimensional schematic depicting the core size (darker grey) and hydrodynamic size (lighter gray) of a QR.

Assuming an "ideal rod" made up of a central cylinder of diameter D_h and height H, sandwiched by two hemispheres of diameter D_h (Figure S16), the hydrodynamic surface area taken up by each ligand (i.e. the ligand footprint, k) can then be calculated by Equation S4-S5 to be k = 1.6 ± 0.6 nm^2 (where A is the hydrodynamic surface area, N is the number of ligands per QR, (i.e. N = 370, see SI Section 2.2), and $H = L_{core} - D_{core}$ = 11.7 ± 5.7 nm). This provides an estimated average glycan density of 0.6 ± 0.2 nm^{-2}. By assuming that the ligands are flexible enough to form a close-packed coverage on the QR surface, then the

hydrodynamic inter-glycan distance, X, can be obtained using Equation S6 to be X = 1.4 ± 0.3 nm, with an inter-glycan angle at the spherical ends of 18 ± 6 ° (Equation S7).

$$A = \pi D_h H + \pi D_h^2 \quad \text{S4}$$

$$k = \frac{A}{N} = \frac{\pi D_h L_h}{N} \quad \text{S5}$$

$$X = 2\sqrt{\frac{k}{\pi}} \quad \text{S6}$$

$$\theta_{end} = 360 \cdot \frac{X}{\pi D_h} \quad \text{S7}$$

In order to compare the protein binding contact area with the dimensions of the glycan-QR, the inter-binding site dimensions for DC-SIGN and DC-SIGNR can be obtained based on the distances between the primary Ca^{2+} ions associated with binding from the models based on 1xar and 1k9i crystal structures of the CRDs.[9,10] This provides DC-SIGNR dimensions of approx. 3.8 × 8.0 nm with an average diagonal primary Ca-Ca distance of ~8.8 nm (Figure S17), and DC-SIGN dimensions of approx. 4.0 × 4.0 nm with an average diagonal primary Ca-Ca distance of ~5.6 nm. These binding contact areas dimensions fall within the dimensions of the both spherical ends and cylindrical middle of QR-DiMan (D_h = 8.9 ± 2.1 nm and H = 11.7 ± 5.7 nm) and easily cover multiple glycans (10-20 DiMan glycans per DC-SIGNR binding contact area). This suggests that neither glycan area nor glycan spacing present the major contribution to the difference in binding mode between the central and end regions of the QR.

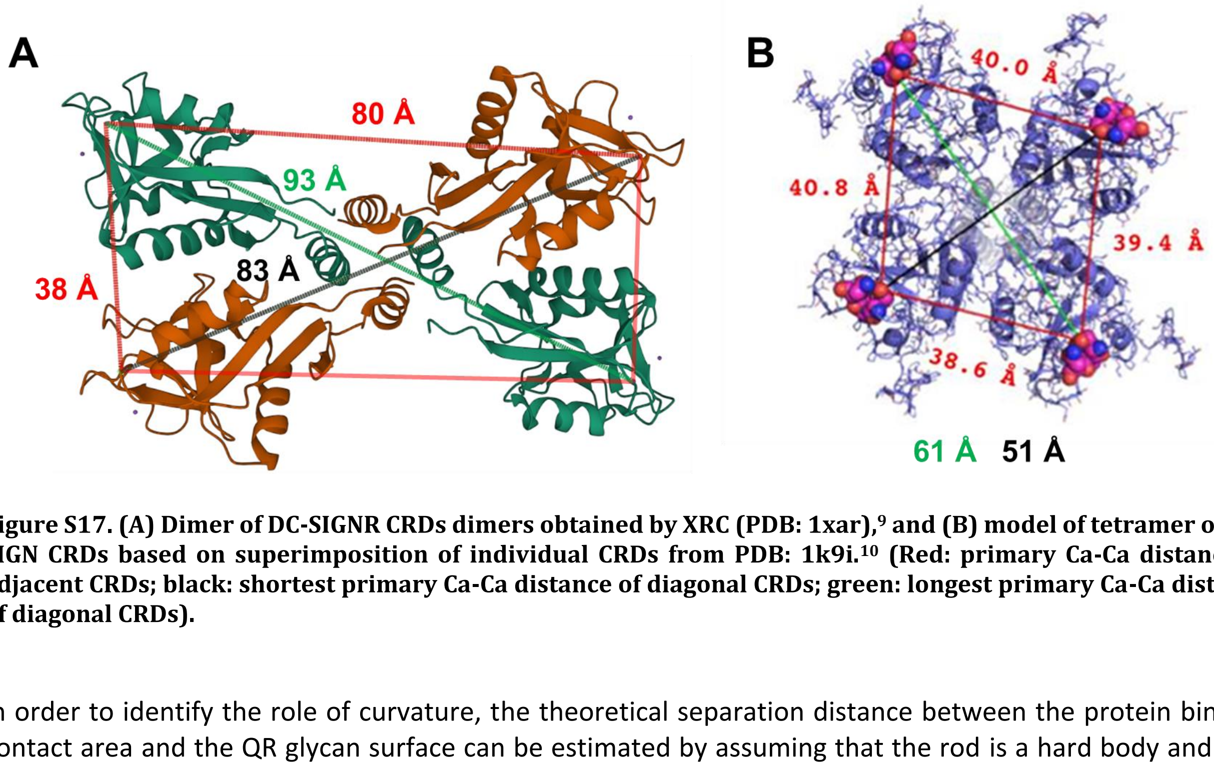

Figure S17. (A) Dimer of DC-SIGNR CRDs dimers obtained by XRC (PDB: 1xar),[9] and (B) model of tetramer of DC-SIGN CRDs based on superimposition of individual CRDs from PDB: 1k9i.[10] (Red: primary Ca-Ca distance of adjacent CRDs; black: shortest primary Ca-Ca distance of diagonal CRDs; green: longest primary Ca-Ca distance of diagonal CRDs).

In order to identify the role of curvature, the theoretical separation distance between the protein binding contact area and the QR glycan surface can be estimated by assuming that the rod is a hard body and that the contact surface of the protein is a rigid plane. Here, the minimum separation distance of the extremities of the contact plane with the hydrodynamic surface of the glycan-QR, d, can be obtained by Equation S8 as shown in Figure S18 (where p is the primary Ca-Ca distance between the binding sites furthest from the QR surface, d is the separation distance and D_h is the hydrodynamic diameter (as depicted in Figure S16). This equation can also be represented in terms of surface curvature, κ, in order to provide a more universal

description of separation distance, where κ is the inverse of the radius of curvature). For DC-SIGNR at the spherical ends, p equals to the diagonal of the contact area which is ~8.8 nm, which equates to a d of ~4 nm. At the cylindrical ends, however, the longer length of DC-SIGNR can align itself along the longer length of the QR meaning only the shorter inter-CRD distance (p = 3.8 nm) contributes to separation distance, leading to a 10-fold smaller d of 0.4 nm. For DC-SIGN, a p of ~5.6 nm leads to a d of ~1 nm. In order to estimate the theoretical separation distance required to enable binding of all CRDs to the glycan surface, the compression length of the LA-EG_{11}-DiMan ligand can be estimated by subtracting the length of the rigid LA substituent (l_{LA} ~0.8 nm; measured with PyMOL) from the average hydrodynamic ligand length ($l = l_{h,ligand} - l_{LA}$ ~ 2.9 – 0.8 = 2.1 nm). The difference between d and l therefore provide a rationale behind the observed differences in binding modes between the two proteins and the two QR regions. For DC-SIGNR, at the spherical ends where $d > l$, the glycan surface is unable to bridge all four CRDs, thus it is must find glycans on neighboring QR-DiMan particles in order to maximally occupy its binding sites. However, at the cylindrical central section $d \ll l$ therefore the glycans only need to be compressed slightly to bridge all 4 of DC-SIGNR's binding sites, allowing for simultaneous binding. For DC-SIGN, $d < l$ at any point on the QR, thus all 4 binding sites can be bridged by the glycan surface irrespective of the QR region, resulting in the observation of only simultaneously bound individual QRs.

$$d = \frac{D_h - \sqrt{{D_h}^2 - p^2}}{2} = \frac{2 - \sqrt{4 - p^2\kappa^2}}{2\kappa} \qquad \text{S8}$$

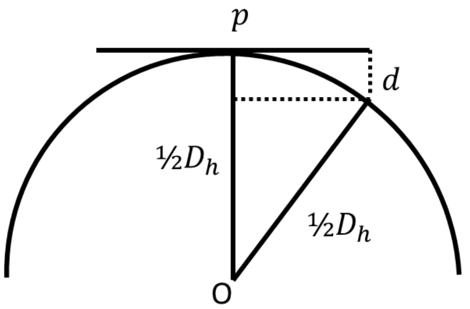

Figure S18. Side-on schematic demonstrating separation distance between the lectin binding contact area to the glycan-QR hydrodynamic surface (d) for a rod of hydrodynamic diameter of D_h with respect to the inter-binding site distance furthest from the rod surface (p). Where d can be obtained by equation S8.

References

(1) Budhadev, D.; Poole, E.; Nehlmeier, I.; Liu, Y.; Hooper, J.; Kalverda, E.; Akshath, U. S.; Hondow, N.; Turnbull, W. B.; Pöhlmann, S.; Guo Y.; Zhou, D. *J. Am. Chem. Soc.* **2020**, *142*, 18022-18034.

(2) Guo, Y.; Nehlmeier, I.; Poole, E.; Sakonsinsiri, C.; Hondow, N.; Brown, A.; Li, Q.; Li, S.; Whitworth, J.; Li, Z.; Yu, A.; Brydson, R.; Turnbull, W. B.; Pöhlmann, S.; Zhou, D. *J. Am. Chem. Soc.* **2017**, *139*, 11833-11844.

(3) Guo, Y.; Sakonsinsiri, C.; Nehlmeier, I.; Fascione, M. A.; Zhang, H.; Wang, W.; Pöhlmann, S.; Turnbull W. B.; Zhou, D. *Angew. Chem. Int. Ed.* **2016**, *55*, 4738-4742.

(4) Wang, W.; Guo, Y.; Tiede, C.; Chen, S.; Kopytynski, M.; Kong, Y.; Kulak, A.; Tomlinson, D.; Chen, R.; McPherson, M.; Zhou, D. *ACS Appl. Mater. Interfaces* **2017**, *9*, 15232-15244.

(5) Saha, S. K.; Brewer, C. F. *Carbohydr. Res.* **1994**, *254*, 157-167.

(6) Hooper, J.; Liu, Y.; Budhadev, D.; Ainaga, D. F.; Hondow, N.; Zhou, D.; Guo, Y. *ACS Appl. Mater. Interfaces* **2022**, *14*, 47385–47396.

(7) Zhou, D.; Li, Y.; Hall, E. A. H.; Abell, C.; Klenerman, D. *Nanoscale* **2011**, *3*, 201-211.

(8) Charbonnière, L. J.; Hildebrandt, N. *Eur. J. Inorg. Chem.* **2008**, *2008*, 3241-3251.

(9) Feinberg, H.; Guo, Y.; Mitchell, D. A.; Drickamer K.; Weis, W. I. *J. Biol. Chem.* **2005**, *280*, 1327-1335.

(10) Tabarani, G.; Thépaut, M.; Stroebel, D.; Ebel, C.; Vivès, C.; Vachette, P.; Durand, D.; Fieschi, F. *J. Biol. Chem.* **2009**, *284*, 21229-21240.
